# How will climate change pathways and mitigation options alter incidence of vector-borne diseases? A framework for leishmaniasis in South and Meso-America

**DOI:** 10.1371/journal.pone.0183583

**Published:** 2017-10-11

**Authors:** Bethan V. Purse, Dario Masante, Nicholas Golding, David Pigott, John C. Day, Sergio Ibañez-Bernal, Melanie Kolb, Laurence Jones

**Affiliations:** 1 NERC Centre for Ecology and Hydrology,Crowmarsh Gifford, Oxfordshire, United Kingdom; 2 NERC Centre for Ecology and Hydrology, Environment Centre Wales, Bangor, United Kingdom; 3 School of BioSciences, University of Melbourne, Victoria, Australia; 4 Institute for Health Metrics and Evaluation, University of Washington, Seattle, Washington, United States of America; 5 Instituto de Ecología, A.C. (INECOL), Red Ambiente y Sustentabilidad, Xalapa, Veracruz, Mexico; 6 Institute of Geography, National Autonomous University of Mexico, Mexico City, Mexico; Johns Hopkins University Bloomberg School of Public Health, UNITED STATES

## Abstract

The enormous global burden of vector-borne diseases disproportionately affects poor people in tropical, developing countries. Changes in vector-borne disease impacts are often linked to human modification of ecosystems as well as climate change. For tropical ecosystems, the health impacts of future environmental and developmental policy depend on how vector-borne disease risks trade off against other ecosystem services across heterogeneous landscapes. By linking future socio-economic and climate change pathways to dynamic land use models, this study is amongst the first to analyse and project impacts of both land use and climate change on continental-scale patterns in vector-borne diseases. Models were developed for cutaneous and visceral leishmaniasis in the Americas—ecologically complex sand fly borne infections linked to tropical forests and diverse wild and domestic mammal hosts. Both diseases were hypothesised to increase with available interface habitat between forest and agricultural or domestic habitats and with mammal biodiversity. However, landscape edge metrics were not important as predictors of leishmaniasis. Models including mammal richness were similar in accuracy and predicted disease extent to models containing only climate and land use predictors. Overall, climatic factors explained 80% and land use factors only 20% of the variance in past disease patterns. Both diseases, but especially cutaneous leishmaniasis, were associated with low seasonality in temperature and precipitation. Since such seasonality increases under future climate change, particularly under strong climate forcing, both diseases were predicted to contract in geographical extent to 2050, with cutaneous leishmaniasis contracting by between 35% and 50%. Whilst visceral leishmaniasis contracted slightly more under strong than weak management for carbon, biodiversity and ecosystem services, future cutaneous leishmaniasis extent was relatively insensitive to future alternative socio-economic pathways. Models parameterised at narrower geographical scales may be more sensitive to land use pattern and project more substantial changes in disease extent under future alternative socio-economic pathways.

## Introduction

Vector-borne diseases have globally significant impacts on public health and socio-economic development. They make up around one-sixth of the illness and disability worldwide, causing an estimated one billion human infections and one million human deaths each year [1]. Recent changes in vector-borne disease patterns have been attributed to wide-ranging drivers, including climate change, increasing rates of international trade and travel and human modification of ecosystems through intensive farming, dams and irrigation, deforestation, human settlement and urbanisation [2, 3].

The current global burden of vector-borne diseases falls disproportionately onto poor populations in tropical, developing countries [4], and exposure is often linked to specific human uses of ecosystems at the landscape level [3, 5]. Pathogens, hosts and vectors involved in vector-borne disease transmission cycles are all environmentally sensitive and vary in their associations with intact natural ecosystems. Impacts of ecosystem changes on vector-borne incidence thus depend on a complex interplay between ecological factors such as the ratio of competent to non-competent hosts and vectors (determined by species-specific responses to biodiversity or ecosystem change) [6] and social factors that determine the contact rates between humans and infected vectors [7]. This interplay is highly context dependent—in the terminology of the ecosystem service concept, ecosystems may provide the “disservice” of maintaining transmission cycles which may lead to infection of humans and/or the “service” of regulating those cycles and controlling spillover into human populations [3]. Tropical ecosystems are under huge pressure from competing land uses and deforestation [8]. They play a critical role in supporting the livelihoods and economic development of large poor communities [9] but also in climate mitigation since they are key terrestrial carbon stores and biodiversity hotspots [10]. For tropical ecosystems then, understanding trade-offs between vector-borne disease “disservices” or “services” and other ecosystem services across heterogeneous landscapes [11] is critical for (i) prediction of health impacts of environmental and development policies that may alter ecosystems (ii) development of decision-support tools to target such measures [4, 12].

A first step in understanding disease-ecosystem linkages is to integrate land use patterns, ecosystem and social factors into interpretations of past disease patterns at a range of geographical scales [13]. Conditional on high explanatory power for past disease patterns, this can facilitate the subsequent integration of these factors into projections of future disease risk given alternative environmental and development policies, as recommended by [4]. Though past vector-borne disease impacts are now quite routinely linked to non-climatic as well as climatic drivers [14, 15], projections of future change in risk for specific diseases are still restricted to climate change scenarios and neglect future socio-economic and public health changes [4] [16] (but see [17]). Furthermore, projections tend to focus on temperature changes rather than precipitation changes, that are easier to represent in global climate models and in terms of mechanistic effects on disease transmission and are rarely validated with observed disease patterns [4]. This study is one of the first to project future vector-borne disease patterns at a continental scale, accounting for future policy-driven changes in land use as well as climate.

Leishmaniasis is transmitted by the bite of Phlebotomine sandflies and is caused by obligate parasite protozoa of the genus *Leishmania* [18]. These parasites have complex life cycles involving multiple insect vectors and mammalian reservoir species [18, 19]. Infection results in wide ranging symptoms, dependent on *Leishmania* species, with the two main outcomes being cutaneous leishmaniasis (CL, 0.7–1.2 million annual cases, [20]) where skin lesions develop around the bite site and visceral leishmaniasis (VL, 0.2–0.4 million annual cases, [20]) that affects the spleen, liver and other lymphoid tissues and can be fatal if left untreated. A particularly debilitating cutaneous form is mucosal or mucocutaneous leishmaniasis (~4% of cutaneous cases in the Americas [21]) which attacks the mucous membranes of the nose, mouth and throat cavities and surrounding tissues and can cause mutilation, disabilities and livelihood impacts if not treated in a timely manner. Collectively, the leishmaniases have huge impact, ranking ninth amongst individual infectious disease burdens globally, with around a 10% death rate [20]. In particular, VL ranks third amongst vector-borne diseases in the number of Disability Adjusted Life Years (number of years lost due to ill-health, disability or early death from a disease) attributed to it (2005 and 2013, [22])

In the Americas, VL is linked with zoonotic peri-domestic cycles with dogs as the main reservoir and foxes and jackals as a sylvatic reservoir [23] [24]. CL is linked to zoonotic, sylvatic cycles involving a wide diversity of wild mammal reservoirs (rodents and large mammals) and sandfly species [19, 23]. Socio-economic factors and ecosystem changes have been linked to impacts of both disease forms, including urbanisation, deforestation, agricultural intensification (dams and irrigation, new crops), human settlement (including migration from rural to urban areas), poverty [15] and marginalisation [23, 25–30]. The precise roles of wild and domestic hosts and different sandfly species in transmission is unquantified for most *Leishmania* parasites and contexts [18, 19]. However, Wood et al. [6] cite the New World *Leishmania* species causing the cutaneous disease form (e.g. *Leishmania braziliensis*, *Leishmania guayanensis*, *Leishmania*. *panamensis*), as examples of parasites that are associated geographically with undisturbed ecosystems because they depend on reservoir mammal hosts that are more abundant in undisturbed than in disturbed habitats. By contrast, the visceral leishmaniasis parasite in the Americas, *Leishmania infantum*, was originally a zoonotic rural infection but has spread into urban and peri-urban areas because of both domestic dog reservoirs and the adaptable sandfly vector, *Lutzomyia longipalpis*, can utilise anthropogenic habitats [25]. Wood et al. [6] thus predict that cutaneous leishmaniasis impacts will be positively associated with intact ecosystems and biodiversity whilst the converse will be true of visceral leishmaniasis.

Working with wide-ranging stakeholders, detailed socio-economic policy scenarios have been developed for this region, encompassing weak versus strong management for carbon, biodiversity and ecosystem services, and mapped geographically from 2005 to 2050 using dynamic land use modelling [31, 32]. This provides us with the opportunity to understand the role of land use and ecosystem patterns alongside climate as drivers of two high-impact vector-borne diseases linked to tropical forests.

The aim of this study is to develop a novel framework for understanding vector-borne disease services and disservices in an ecosystem context by:

- Quantifying the relative role of climate, land use and ecosystem patterns, and mammal biodiversity in driving past distributions of cutaneous and visceral leishmaniasis
- Ranking potential socio-economic and policy scenarios, with weak and strong management for carbon, biodiversity and ecosystem services, and climate pathways in terms of likely disease disservices from the leishmaniases

We hypothesise that human disease occurrence will be more likely where forest habitats are fragmented by agricultural intensification or urbanisation, since this would either (i) newly expose people to the leishmaniasis pathogen pool circulating in sylvatic cycles or (ii) perturb pathogen-vector-host dynamics in the system, enhancing cross-species transmission rates (two alternative mechanisms for impacts of land use change on disease emergence set out in [7]). We expect the probability of leishmaniasis occurrence to increase with the availability of human-modified habitats (crops, grazing, urban areas) and the density of forest and crop edges (edge density metrics), indicative of interface habitats within which human exposure could occur [23]. We expect these effects to be stronger for CL than VL given the wider range of wild mammal reservoir species incriminated in transmission [23]. For similar reasons, we expect CL, to be particularly influenced by mammal biodiversity.

## Materials and methods

### Leishmaniasis distribution data

**Fig 1:**
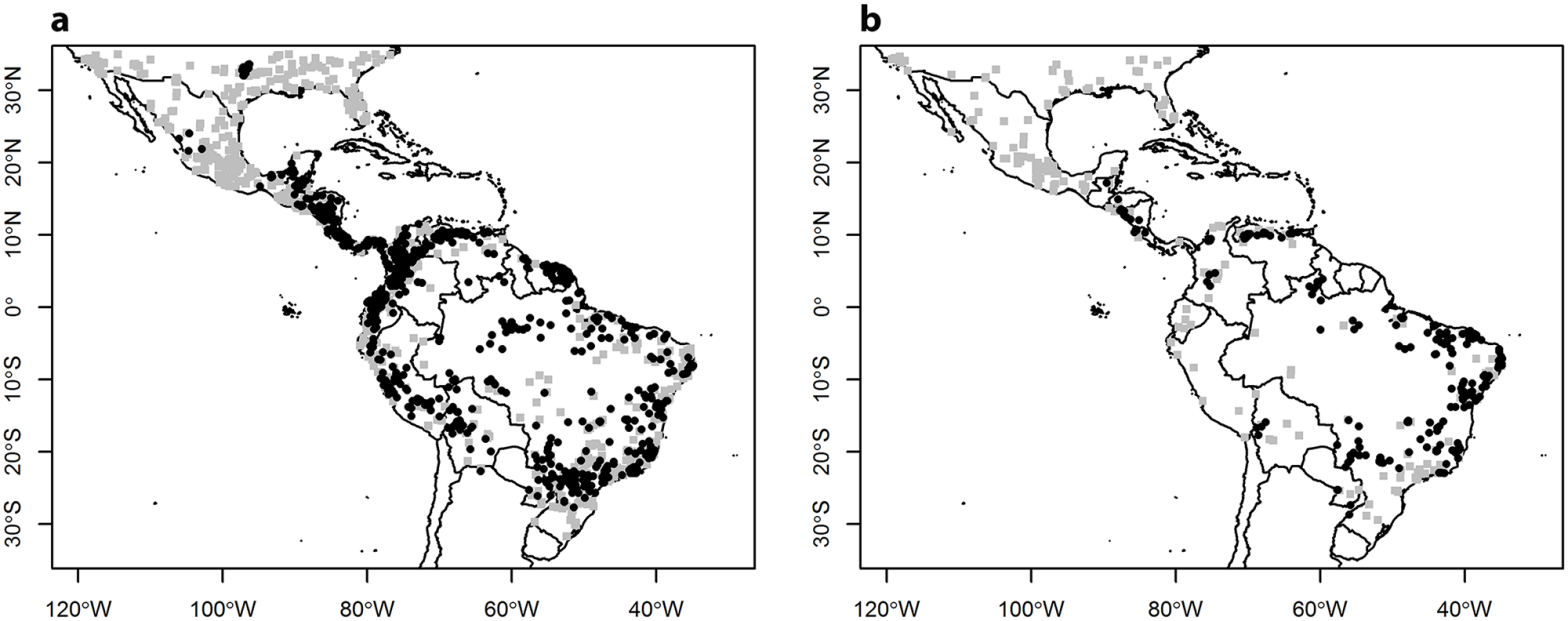
Map of study area indicating the distributional data for the leishmaniases (black circles) used to parameterise environmental models (a) cutaneous leishmaniasis (n = 803 squares) (b) visceral leishmaniasis (n = 201 squares). Grey squares indicate the locations of pseudo-absence data points for one iteration of the model. Tick marks on the x and y axes indicate degrees latitude and longitude respectively and the study grain is 5 arc minute squares.

For South and Meso America (including Belize, Bolivia, Brazil, Colombia, Costa Rica, Ecuador, El Salvador, French Guiana, Guatemala, Guyana, Honduras, Mexico, Nicaragua, Panama, Paraguay, Peru, Suriname, Venezuela), point level occurrence of CL, (3338 records available) and VL (859 records available) cases were obtained from the dataset compiled by [33]. This included data from literature sources, from the HealthMap database (http://healthmap.org/en/), from GenBank and Centre National de Référence des Leishmanioses (CNR-L) in Montpellier, France. Disease presence was summarised at the study grid-square resolution of 5 arc minutes (approximately 10 by 10km squares at the equator), giving 201 presence cells for VL and 803 presence cells for CL (Fig 1). The resulting observed disease distribution was, therefore, independent of disease burden or case density. The dataset spans the period between 1970 and 2013, but the vast majority of squares (73.8% for CL and 74.1% for VL) in the disease distribution have been recorded recently, since 1990.

CL and VL records from Brazil, between 2008 and 2011, were used to obtain an independent estimate of disease incidence for model validation. These records of infection, at a municipality level, were obtained from the Brazilian Ministry of Health; the Sistema de Informação de Agravos de Notificação (SINAN, http://dtr2004.saude.gov.br/sinanweb/) reporting network. The average number of infections over these four years was calculated for each municipality and divided by population size for 2000 from The Gridded Population of the World version 3 (GPW3) population density database [34].

### Socio-economic and climate change scenarios

**Fig 2:**
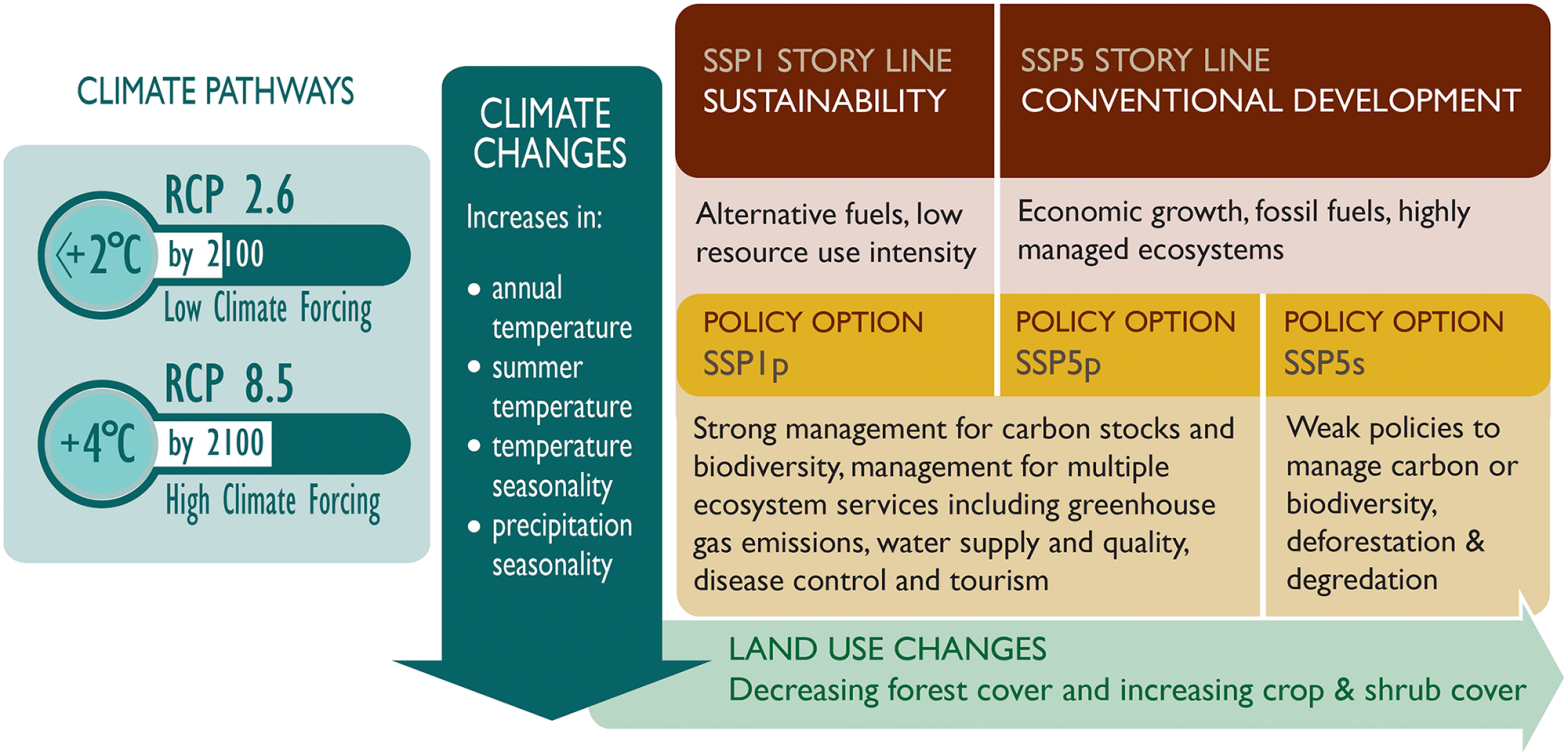
The trajectory of changes in climate and land use under alternative future socio-economic storylines, climate change pathways and policies. A matrix of six alternative scenarios is depicted, 3 SSPs in columns x 2 climate change pathways in rows. Each scenario was realised in terms of mapped values of cover of CLUE land use categories and Worldclim climate variables in 2005 (to reflect the recent past) and 2050.

We considered a matrix of six alternative future scenarios organised around two important dimensions: the extent of climate change and possible future socio-economic conditions (Fig 2). The Shared Socio-economic Pathway [35] framework depicts five different global futures (SSP1-5) with different socio-economic conditions, reflecting different socio-economic challenges to climate change mitigation and adaptation and encompassing possible trends in agriculture and land use. Here, for Meso and South America, we developed storylines under two SSPs; SSP1 represents Sustainability where there are sustained efforts to reduce resource use intensity and fossil fuel dependency; SSP5 represents Conventional development, in which the world focuses on economic growth and the energy system is dominated by fossil fuels (rather than biofuels for example) and ecosystems are highly managed [31]. Within each SSP, policies to manage carbon stocks and biodiversity can be strong (SSP1p and SSP5p), accounting for multiple ecosystem services, or weak, with high rates of deforestation and degradation (SSP5s), making three alternative SSP/policy combinations in the matrix. The two contrasting climate forcing scenarios in the matrix were the representative concentration pathways (RCPs) RCP 2.6 and RCP 8.5 [36, 37].

Land use change under alternative SSP/policy combinations was modelled using the CLUE (Conversion of Land Use and its Effects) model, which dynamically simulates competition between land use types under different drivers of land use change [32]. This model was applied to the study area at a grid-square resolution of 30 arc seconds. Scenarios of future urban expansion were not considered, and the urban land cover was treated as a static land use category. Data for climate conditions under the RCPs were obtained from Worldclim 2050 projections for HadGEM2-ES global circulation models (www.worldclim.org) at a resolution of 5 arc minutes (0.0833 degrees latitude/longitude) for the climate predictors given in Table 1. Fig 2 summarises the trajectories of changes in land use and climate variables under the different climate change pathways and SSP and policy combinations (see Supplementary Information S2 File). The year 2005 represented the recent past, with all scenario combinations run to 2050.

**Table 1:** Potential environmental predictors of leishmaniasis distribution.

Category	Variable description (abbreviation)
Climate	annual mean temperature (bio1)
Climate	annual precipitation (bio12)
Climate	precipitation seasonality (bio15)
Climate	precipitation of driest quarter (bio17)
Climate	temperature seasonality (bio4)
Climate	maximum temperature of warmest month (bio5)
Landscape	mean elevation (across 30 arc second cell in 5 arc minute cell) (elevation)
Landscape	Forest cover (forest cover) and fragmentation (forest edge)
Landscape	Flooded /wetland cover
Landscape	shrubland cover
Landscape	cover of cropland linked to food, feed and fibre products (crop FoodFeedFiber)
Landscape	shrubland grazed cover
Landscape	grassland grazed cover
Landscape	grasssland cover
Landscape	cover and fragmentation of cropland linked to perennial food crops FoodPerennial cover (crop FoodPerennial cover and crop FoodPerennial edge)
Landscape	sparse grazed cover
Landscape	sparse cover
Landscape	urban cover
Landscape	cover and fragmentation of energy crops (crop energy cover and crop energy edge)
Landscape	Global Irrigated Area
Host	mammal species richness
Host	Rodentia richness
Host	Primate richness
Host	Marsupial richness
Host	Carnivoria richness
Host	Chiroptera richness

### Environmental predictors

A prerequisite to predicting the impact of future scenarios on leishmaniasis distribution was to establish the strength of relationships between leishmaniasis and environmental factors in the recent past. For both the 2005 and 2050 time points, a suite of gridded, biologically plausible, correlates was generated within climate, landscape and host categories.

#### Climate

Temperature and precipitation have been found previously to be key predictors of past sand fly and disease distributions [15, 30, 38–40]. Humidity and moisture levels determine the availability of breeding and resting sites of phlebotomine sandflies [18] whilst temperature affects the development of the *Leishmania* parasite inside the sand fly [41, 42] as well as key sand fly life cycle parameters, including development and emergence rates, oviposition, metabolism and adult mortality [18]. Six uncorrelated (r < 0.8) WorldClim seasonal variables were selected amongst the nine used previously to model sand fly and Leishmaniases distributions [38, 39] (Table 1).

#### Landscape

Around 60 sand fly vector species are known to be involved in the transmission of multiple *Leishmania* pathogens to humans in South and Meso America [18] [43]. Though some sandfly vectors are forest specialists, other species utilise human-modified habitats including cropland, coffee plantations, shrubland and peri-urban areas [44, 45]. Given this inter-specific variability in habitat associations, we examined the importance of availability and fragmentation of a wide range of CLUE land use classes that could contain sandfly and human habitats and might be impacted by different climate mitigation options (Table A in S1 File). Fragmentation metrics such as the length of edge between anthropogenic and natural habitats are increasingly being linked to higher vector-borne disease incidence or occurrence [46, 47] because they reflect the amount of interface habitat where human exposure to pathogens occurs. The full rationale for selection among CLUE classes and processing of these fragmentation metrics is described in Supplementary Information S1 File.

#### Hosts

Human population density and urban land cover was considered as a predictor because (i) for a subset of *Leishmania* pathogens, humans may function as reservoir of infection rather than an incidental hosts [48]; (ii) for the visceral leishmaniasis pathogen, *L*. *infantum*, domestic dogs are important reservoirs [25, 48]. Since data on dog densities are not available at a continental scale, we assume the incidence of dogs to scale with the incidence of human populations at this study grain. The GPW3 human population density data and the Urban Extents surface from the Global Rural–Urban Mapping were highly correlated with the CLUE urban cover class, therefore only the latter predictor was included in models. The fact that human case reporting may be less likely in rural areas with poor access to health care is accounted for in the model pseudo-absence selection procedure (see later).

Sylvatic wild mammal species, spanning several orders, are widely implicated as reservoirs of the *Leishmania* species that cause cutaneous leishmaniasis [19]. For the *Leishmania* species causing visceral leishmaniasis in peri-domestic settings, it is possible that wild mammals provide a common source of infection to humans and dogs [19, 25]. Since the roles of wild mammal species and assemblages in transmission are important but poorly quantified, and spatial population data for individual wild mammal species are lacking at a continental scale, we included available spatial patterns in mammal richness [49] as a predictor. Recent past models were developed and compared using three suites of predictors: 1) abiotic (climate + land use) only 2) abiotic + richness of all mammal species 3) abiotic + richness of individual mammal orders (Primata, Chioptera, Marsupialia, Rodentia, Carnivora) that either have high numbers of species already incriminated as reservoirs or that have high ecological diversity and overlap with human habitation.

### Modelling leishmaniasis distributions with boosted regression trees

A boosted regression tree (BRT) modelling [50, 51] framework was used to determine the sensitivity of recent occurrence of CL and VL to land use, climate and host variability, and to generate maps of recent distributions and future distributions under socio-economic policy and climate change scenarios. BRTs combine regression trees, which build a set of decision rules on the predictor variables by portioning the data into successively smaller groups with binary splits [50, 51], and boosting, which selects the tree that minimises the loss of function, to best capture the variables that define the distribution of the input data. BRTs have been shown to have high performance amongst methods used to predict species distributions [52], probably due to their ability to fit complex, non-linear responses to environmental covariates.

Since boosted regression trees require both presence and absence data, a strategy was developed for selecting pseudo-absence data. Since, human cases were likely to be recorded more intensively in urban than in rural areas, to mimic the process by which presence data are generated, the selection of pseudo-absences (equal in number to the presences after [53]) was weighted in proportion to human density values in the GPW3 layer. Twenty different input datasets were generated by repeating this process, and twenty BRT sub-models were fitted using the gbm.step function of the dismo package in R [54]. Model settings were as follows: learning rate = 0.05, tree complexity = 4, bag rate = 0.5. The gbm.step function automatically identifies the optimum number of trees for a BRT model using ten-fold cross-validation, selecting the number of trees that minimises hold-out deviance (cross-validation deviance) across folds. In addition to the cross-validation deviance, gbm.step reports several metrics of model performance in cross-validation across folds including (i) the Area Under the Receiver Operator Curve Statistic (AUC) on hold-out dataset [55], or cross-validation AUC, where an AUC value of 0.5 indicates no discriminative ability between presence and absence, and a value of 1 indicates perfect discrimination; (ii) cross-validation coefficient, which is the Pearson’s correlation coefficient between the predicted probability of presence and the true presence/background for the hold-out dataset. We report the average of these metrics across the twenty BRT sub-models. It should be noted that since the models are parameterised with presence-only data and the true prevalence of the disease across the study region is unknown, they predict the relative rather than absolute probability of presence between cells.

Relative contribution statistics of predictor variables are reported only for the BRT model with the optimum number of trees (not for the folds). Relative importance is defined as the number of times a variable is selected for splitting, weighted by the squared improvement to the model as a result of each split and averaged over all trees [56]. These contributions are scaled to sum to 100, with a higher number indicating a greater effect on the response. Again we report the average of these values across the twenty BRT sub-models. This model fitting process was repeated for three different suites of environmental predictors as above.

To predict the distribution of leishmaniases under recent past and future land use and climate change scenarios, each BRT-submodel (from the abiotic-only suite of predictors) was applied to the prediction layers for a given land use and climate combination using the predict.gbm function of the gbm package [57] in R. The predicted relative probability of presence was averaged across sub-models to produce an ensemble relative probability of presence (and standard deviation). External validation of recent past models was achieved by correlating the average case rate per municipality (2007–2011, see above) with the average predicted relative probability of presence across pixels in that municipality for each disease form. For each BRT model, the threshold relative probability of presence that maximises discrimination between presence/background for the hold-out dataset was calculated by gbm.step and averaged across folds (cross-validation threshold). Occurrence patterns were compared between the recent past and the future predicted distributions by converting the recent past and future predicted distributions to binary presence-absence maps per sub-model using the mean cross-validation threshold relative probability of presence for each recent past sub-model. The predicted extent of occurrence in terms of number of study grid squares or pixels was calculated for the recent past and each future land use and climate combination. The geographical extent of predictions was limited to the region for which the environmental predictors were available (the most restrictive of these being the land-cover metric) and of necessity omitted some adjacent areas of autochthonous leishmaniasis transmission such as Argentina. As per the matrix in Fig 2, six different combinations of scenarios were considered i.e. 2 RCPs x 3 SSPs x 1 circulation model (HadGEM2-ES).

## Results

### Environmental predictors of the recent past distribution of leishmaniases

Tables 2 and 3 identify the top ten predictor variables for CL and VL respectively when abiotic variables (here defined as climate and landscape) are considered alone or in combination with mammal richness predictors. In the abiotic only models (left-hand columns, Tables 2 and 3), for both disease forms, climatic predictors explain around 80% of the variance in distribution and landscape predictors around 20%. For CL, temperature seasonality and precipitation seasonality were key predictors, jointly explaining over 40% of the variance in distribution. CL occurrence was favoured at low to medium levels of seasonality in both temperature and precipitation. For VL, annual mean temperature and temperature seasonality explained over 40% of the variance in distribution and occurrence was favoured under warm annual temperature conditions with low seasonality. The most important landscape predictor for CL was elevation followed by grazed grassland cover, perennial food crop cover and the amount of irrigated land. For VL, the amount of irrigated land was the key landscape predictor followed by elevation and grazed grassland cover. The edge landscape metrics did not rank amongst the ten most important predictors for either disease form.

**Table 2:** Percentage contribution of top ten ranked predictors to models of cutaneous leishmaniasis where different sets of predictors were considered (averaged across 20 sub-models).

*Abiotic predictors only*			*Abiotic predictors and all mammal richness*			*Abiotic predictors and mammal order richness*		
Predictor	mean	sd	Predictor	mean	sd	Predictor	mean	sd
temperature seasonality(bio4)	31.4	2.3	richness (all mammals)	23.0	2.9	richness primates	18.6	2.2
precipitation seasonality(bio15)	10.4	1.5	temperature seasonality(bio4)	17.2	2.0	temperature seasonality(bio4)	10.3	1.1
elevation	8.1	1.0	precipitation seasonality(bio15)	7.4	1.0	richness Chiroptera	8.0	1.3
precipitation driest quarter (bio17)	6.6	1.4	elevation	6.4	1.0	richness marsupials	7.9	1.3
temperature annual mean (bio1)	5.8	0.7	max. temp. warmest month (bio5)	6.0	1.0	precipitation annual mean (bio12)	6.4	1.6
precipitation annual mean (bio12)	5.7	0.8	temperature annual mean (bio1)	5.2	0.7	richness Carnivora	6.2	0.9
max. temp. warmest month (bio5)	4.9	0.9	precipitation driest quarter (bio17)	4.8	0.9	precipitation seasonality(bio15)	4.6	0.7
grazed grassland cover	4.0	0.6	precipitation annual mean (bio12)	4.6	0.6	elevation	4.4	0.5
cropland foodperennial	3.5	0.9	grazed grassland cover	4.0	0.7	max. temp. warmest month (bio5)	3.9	0.6
Irrigated land area	3.0	0.6	cropland foodperennial	2.7	0.7	richness (Rodentia)	3.2	0.6

**Table 3:** Percentage contribution of predictors to models of visceral leishmaniasis where different sets of predictors were considered (averaged across 20 sub-models).

*Abiotic predictors only*			*Abiotic predictors and all mammal richness*			*Abiotic predictors and mammal order richness*		
Predictor	mean	sd	Predictor	mean	sd	Predictor	mean	sd
temperature annual mean (bio1)	28.8	3.4	temperature annual mean (bio1)	26.5	4.8	richness primates	20.6	5.4
temperature seasonality (bio4)	12.7	2.7	richness (all mammals)	17.2	3.6	temperature annual mean (bio1)	17.9	3.9
precipitation annual mean (bio12)	7.2	1.4	precipitation annual mean (bio12)	6.6	1.2	richness marsupials	9.1	2.1
irrigated land area	6.8	2.2	temperature seasonality(bio4)	6.3	1.5	richness Chiroptera	7.1	2.0
elevation	6.0	1.4	grazed grassland cover	5.2	1.5	precipitation driest quarter (bio17)	4.3	1.8
grazed grassland cover	5.7	1.5	elevation	4.8	1.3	precipitation annual mean (bio12)	4.2	1.3
precipitation driest quarter (bio17)	5.2	2.0	precipitation driest quarter (bio17)	4.7	2.0	temperature seasonality (bio4)	3.6	1.0
max. temp. warmest month (bio5)	4.1	0.9	irrigated land area	4.3	1.9	elevation	3.3	0.9
precipitation seasonality(bio15)	3.4	0.8	max. temp. warmest month (bio5)	3.4	1.1	grazed grassland cover	3.3	1.2
forest cover	3.3	1.1	forest cover	3.1	1.2	max. temp. warmest month (bio5)	3.0	1.5

When richness of all mammals is added to the models (middle panel, Table 2), it becomes the most important predictor for CL, explaining 23% of the variance, and the second most important predictor behind annual temperature for VL, explaining 17% of the variance in distribution. The response plots (predicted relative probability of presence plotted as a function of environmental variables, not shown) reveal that the probability of occurrence of both VL and CL increases as mammal richness increases. When the richness of individual mammal orders are added to the models, these again outrank some climate and landscape variables as predictors (right-hand panel, Table 2). However, models incorporating mammal richness variables were not found to have substantially higher accuracy than models excluding mammal richness variables (Table 3), differing in AUC or correlation coefficients values by 0.01 or less, and they produced similar predicted patterns of occurrence (Figure A and B in S3 File).

Internal validation statistics of all models were high—for CL mean AUCs were 0.82 or higher (Table 4a) and the mean correlations were 0.56 or higher. For VL, mean AUC were 0.85 or higher and the mean correlations were 0.63 or higher (Table 4b). Predicted relative probability of presence from models with abiotic predictors showed significant positive correlations with independent municipality level data on the prevalence of CL and VL (CL: r = 0.214, p<0.0001; VL: r = 0.217, p<0.0001).

**Table 4:** Mean accuracy statistics for models of cutaneous and visceral leishmaniasis for models considering only abiotic predictors versus those considering mammal richness alongside abiotic predictors. A. cutaneous leishmaniasis. B. visceral leishmaniasis.

A						
	Abiotic predictors only		Abiotic predictors + all mammal richness		Abiotic predictors + richness mammal orders	
Statistic	Mean	sd	mean	sd	mean	sd
Total deviance	1.39	0.00	1.39	0.00	1.39	0.00
Residual deviance	0.82	0.04	0.79	0.04	0.73	0.03
Cross-validation deviance	1.03	0.02	1.02	0.02	0.98	0.02
Cross-validation correlation	0.56	0.02	0.57	0.02	0.59	0.01
Cross-validation AUC	0.82	0.01	0.82	0.01	0.83	0.01
Cross-validation threshold	0.53	0.01	0.54	0.01	0.56	0.01
B						
	Abiotic predictors only		Abiotic predictors + all mammal richness		Abiotic predictors + richness mammal orders	
Statistic	Mean	sd	mean	sd	mean	sd
Total deviance	1.39	0.00	1.39	0.00	1.39	0.00
Residual deviance	0.53	0.08	0.56	0.06	0.54	0.06
Cross-validation deviance	0.94	0.04	0.93	0.03	0.90	0.04
Cross-validation correlation	0.63	0.03	0.64	0.02	0.65	0.02
Cross-validation AUC	0.85	0.02	0.86	0.01	0.86	0.01
Cross-validation threshold	0.57	0.02	0.58	0.02	0.59	0.02

**Fig 3:**
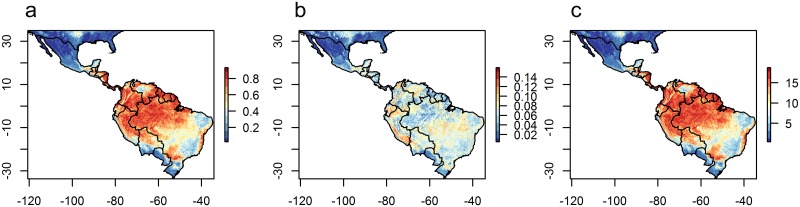
Predicted (a) mean and (b) standard deviation of the relative probability of presence of CL and (c) the sum of times CL is predicted to be present, across 20 runs of models built with abiotic variables only.

**Fig 4:**
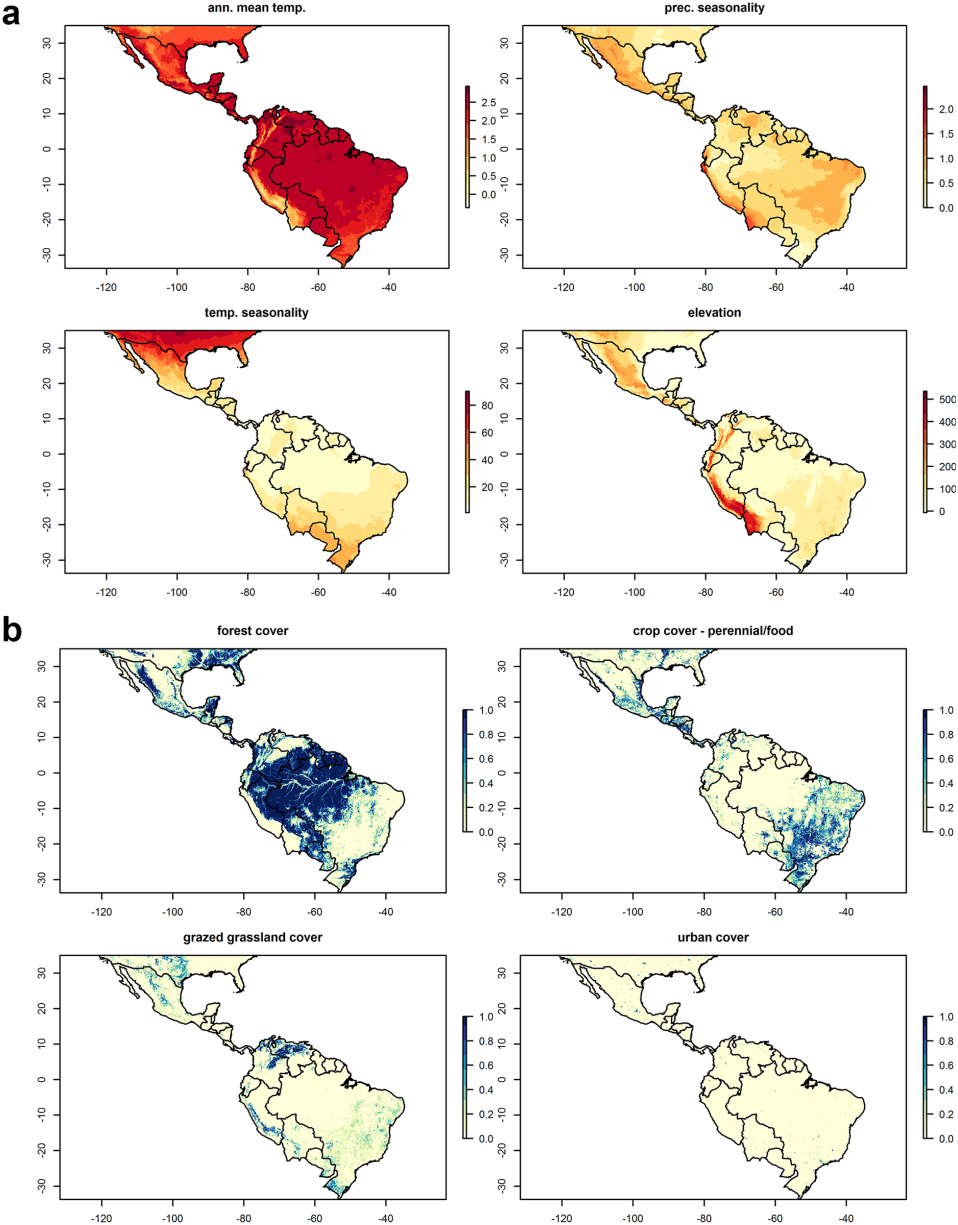
Geographical variation in selected (a) climate and (b) landscape predictors of leishmaniasis distribution in the recent past period (2005).

**Fig 5:**
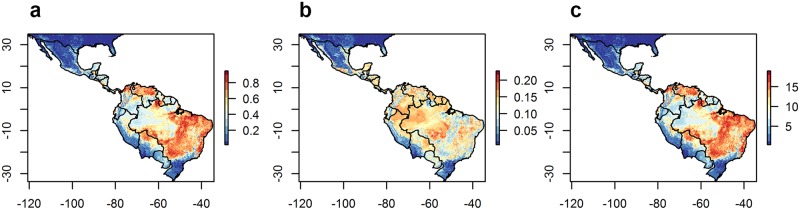
Predicted (a) mean and (b) standard deviation of the relative probability of presence of VL and (c) the sum of times VL is predicted to be present across 20 runs of models built with abiotic variables only.

Though forest cover was not a top predictor in models, the predicted distribution of CL largely coincides with the Amazon Basin and other areas of rainforest (Figs 3 and 4). Additional areas of occurrence are along the east coast and south of Brazil. The highest uncertainty in these predictions is found in the west of the region (Fig 3b). In contrast, VL is predicted to be less extensive and to occur largely in the east of the region, in agricultural areas in east and south Brazil, and in the north along the coast (Fig 5a). The model outputs for VL are more uncertain in areas of predicted absence in the Amazon and other forested areas (Fig 5b). Both disease forms are predicted to be absent from high mountain areas, where annual mean temperatures are low (Fig 4). Compared to predictions from recent global models by Pigott et al. [15], this study predicts both disease forms to occur slightly more widely but in similar geographical hotspots.

When the bias in disease recording towards more populous areas is not accounted for in models, urban land cover is selected as the dominant predictor (Table A in S4 File) and overall land cover effects are of as much importance as climatic effects. However, these uncorrected models vastly under-predict the distributions of both disease forms (Figure A in S4 File) with the corrected models showing greater consistency with predicted distributions from other environmental models and with expert opinion on the geographical variability in cases [15].

### Impact of future land use and climate changes on the geographical extent of leishmaniases

Both representative concentration pathways show increases in annual mean temperature (bio1) (obviously more pronounced in RCP 8.5), mean temperature seasonality (bio4) and maximum temperature of the warmest month (bio 5) (Figure A in S2 File). The area of cropland increases in all scenarios but is most pronounced in the SSP5 scenarios, particularly SSP5s (Figures B to D in S2 File). The amount of forest cover is reduced on average in the SSP 5 land use scenarios but is similar to current day values in SSP 1 (Figure E in S2 File).

**Fig 6:**
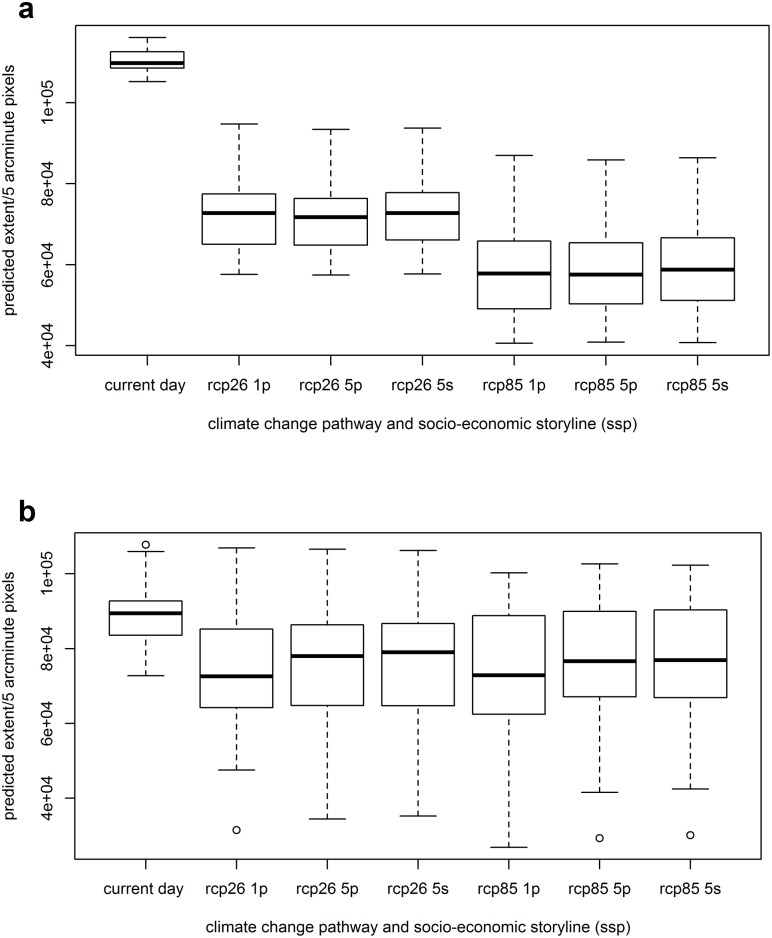
The predicted extent of (a) cutaneous and (b) visceral leishmaniasis under recent past conditions and under alternative climate change pathways and socio-economic storylines. The heavy blacklines across the middle of the box indicate median predicted extent across the 20 model runs; the box indicates the interquartile range of the data whilst the whiskers indicates the extremes. Note that the threshold that best predicted disease presence in cross-validation across model runs was used to assign pixels to presence or absence classes (values in Table 3).

Fig 6 compares the predicted extent of each disease across the whole study region under recent past conditions versus each of the alternative climate change pathways and socio-economic storylines (for the HadGEM2-ES climate model). Both leishmaniasis disease forms are predicted to decrease in extent into the future, possibly due to association of both diseases with low seasonality in both temperature and precipitation (seasonality in both these climate variables increases into the future).

**Fig 7:**
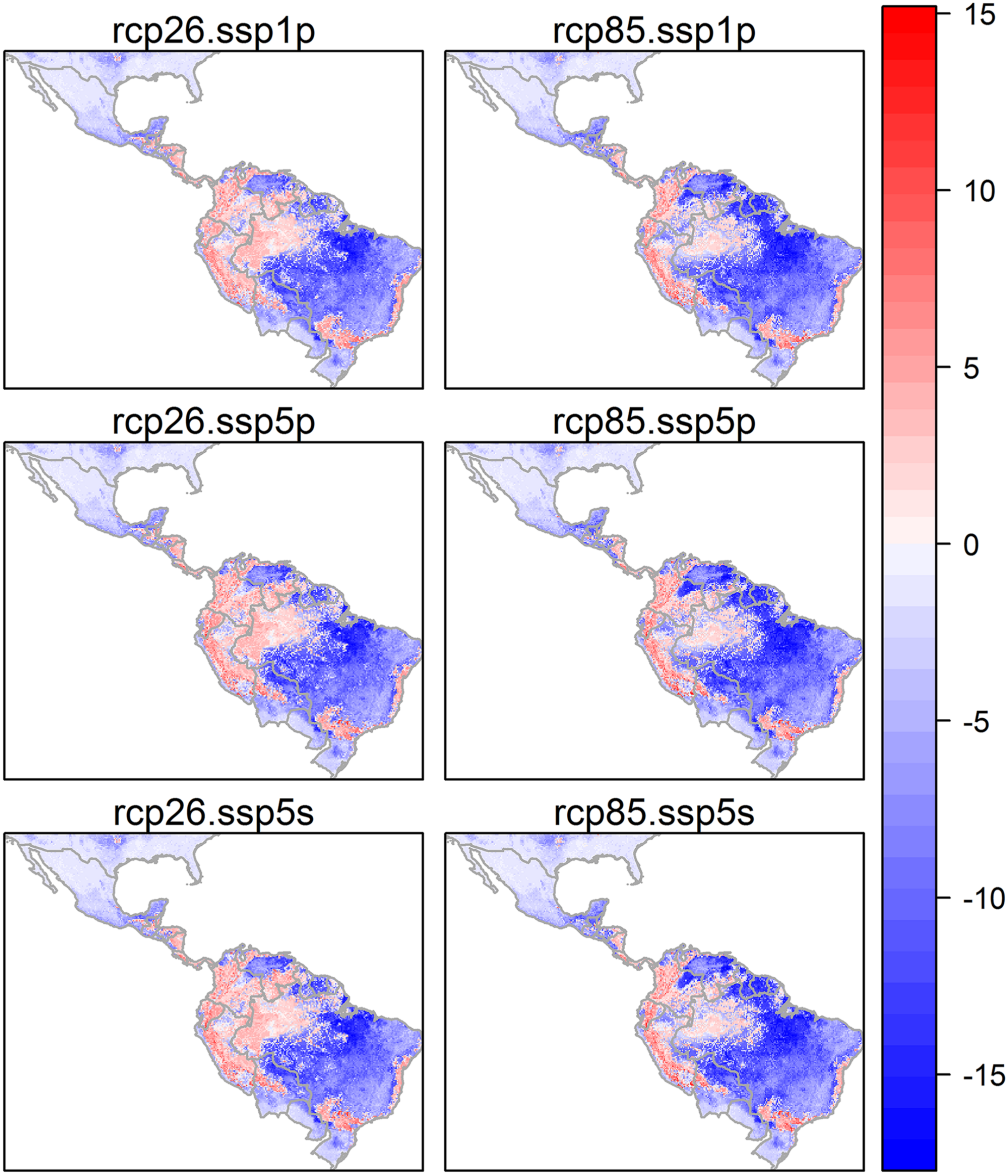
Change in the predicted presence of cutaneous leishmaniasis between the recent past and future (2050) conditions under alternative climate change and socio-economic scenarios. This was calculated by subtracting the number of times a pixel was predicted as present in the recent past (across 20 model runs) from the number of times a pixel was predicted as present in the future. Areas in pink show areas that are likely more favourable for disease and areas in blue show areas that are less favourable for the disease in the future.

For cutaneous leishmaniasis, the geographical extent of disease is predicted to decrease dramatically under all future scenarios, with the extent of decrease being greatest under the rcp 8.5 climate pathway (almost 50% decrease in extent versus 35% under rcp 2.6). Predicted changed extents of CL were relatively insensitive to different socio-economic storylines. Although decreases in extent are predicted on average across the region, some recent presence zones will be maintained under all future scenarios and may even increase slightly in favourability for disease (Figure A in S5 File, Fig 7), namely along the east coast of Brazil and in the west, in Peru, Colombia, Ecuador and west Brazil. Areas of recent presence inland in north and west Brazil are lost in all scenarios (Figure B in S5 File, Fig 7).

**Fig 8:**
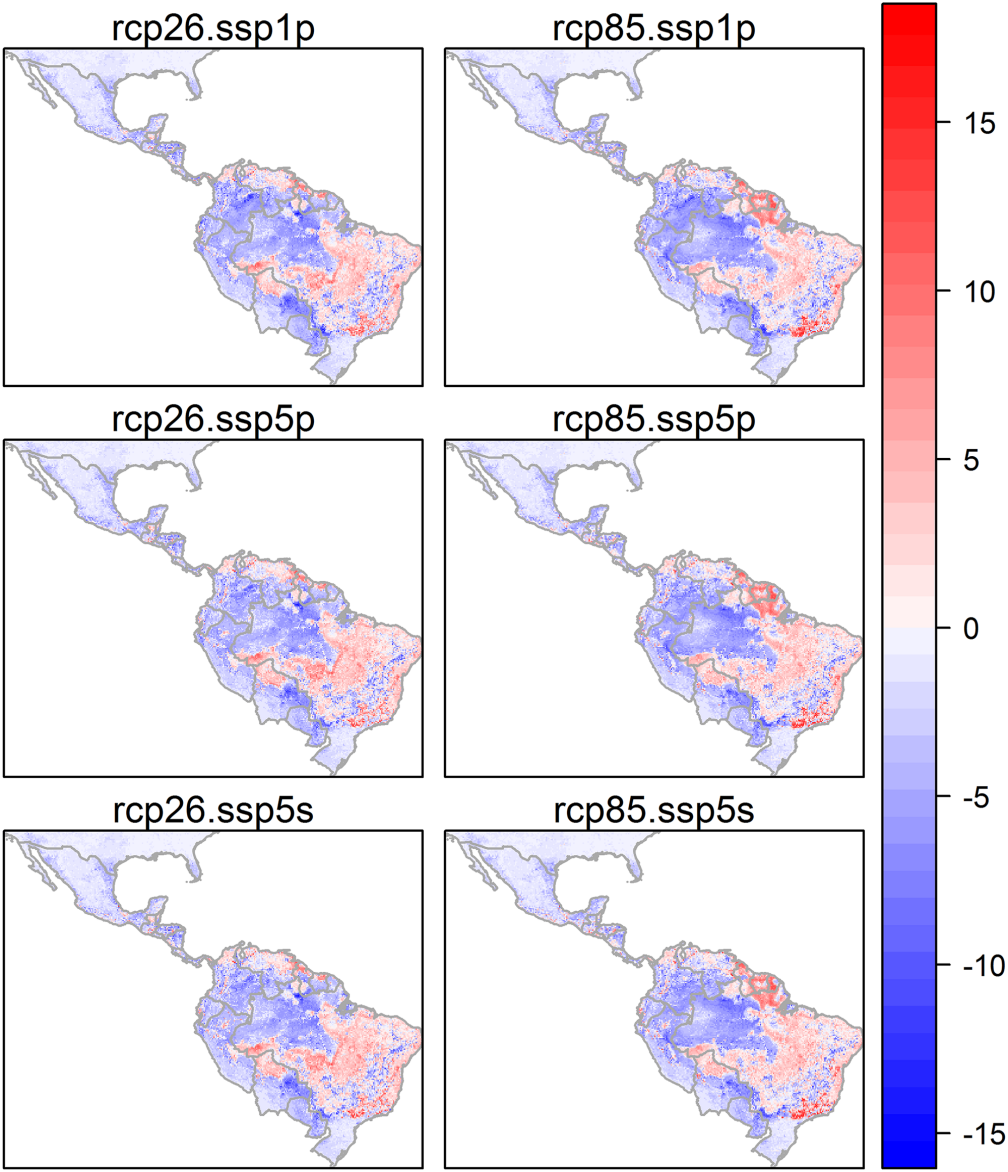
Change in the predicted relative probability of presence of visceral leishmaniasis between the recent past and future (2050) conditions under alternative climate change and socio-economic scenarios. This was found by subtracting the number of times a pixel was predicted as present in the recent past (across 20 model runs) from the number of times a pixel was predicted as present in the future. Areas in pink show areas that are likely more favourable for disease and areas in blue show areas that are less favourable for the disease in the future.

For visceral leishmaniasis, the predicted decreases in extent into the future were less dramatic than for CL, ranging from a 15 to 18% decrease, and were much less sensitive to climate pathway than CL was. VL was predicted to be slightly more extensive under the 5s or 5p storylines than the 1p storyline. geographical distribution of favourable conditions for this disease form are relatively stable between recent past and future scenarios, with stable presence zones in the east of the region, in agricultural areas in east and south Brazil, and in the north along the coast (Fig 8, Figure B in S5 File).

## Discussion

This study advances understanding of the geographical determinants of cutaneous and visceral leishmaniasis in the Americas by considering wide-ranging climate, landscape and ecological risk factors in the same analysis. We provide the first predictions of future change in CL and VL distributions under alternative climate pathways and socio-economic scenarios (strong versus weak management for carbon stock and biodiversity and multiple ecosystem services) by applying disease-environment relationships from the recent past to projected, scenario-specific, climate and land use changes.

Our finding that climate dominates as a driver of recent past patterns in both CL and VL is consistent with widespread findings of significant temperature and precipitation effects on disease and sandflies at similar study grain size (~ 1-10km) [15, 30, 38–40]. It is also consistent with known climatic impacts on sandfly and leishmaniasis life cycles [18]. Few empirical studies have linked disease or sandfly patterns to climate seasonality, but we found both disease forms to be commonest in areas with narrow seasonal ranges of temperature and precipitation [58, 59]. In such areas, immature development and adult survival of sandflies may be less compromised by drying and high temperatures [18].

We hypothesised that disease impacts, especially of cutaneous leishmaniasis, would scale with the fragmentation of forests by agricultural intensification or urbanisation due to the increased availability of interface habitats where human exposure could occur [23]. By contrast, we found that land use factors explained a small proportion (~ 20%) of the variance in disease patterns for both CL and VL at this study grain. Crop types and grazed grassland cover were ranked in the top ten predictors for both diseases but edge metrics did not rank at all amongst top model predictors. Stronger impacts of landscape factors on leishmaniasis patterns, particularly forest proximity, have been detected in studies conducted within smaller geographical regions (single country/district) at a finer spatial grain (municipality or village level surveys) and where the dependent variable (e.g. annual disease incidence) contains information about the extent of disease impact [27, 29, 60]. We attribute the weak landscape effects detected in this study (despite spatially continuous, fine-scale land use data) to the continental study scale and presence-only nature of the available disease data, common to many global infectious diseases [61]. We expect impacts of landscape and social factors to be easier to detect when modelling disease distributions using case incidence or prevalence data that correspond more closely to the intensity of transmission than presence-only data.

When mammal biodiversity was incorporated into models, we found a positive impact of richness on patterns in both leishmaniasis forms. This predictor explained a slightly high proportion of variance in CL than VL distribution, consistent with the stronger links of CL to sylvatic cycles and a diversity of wild mammal species [23]. This finding initially seems consistent with the prediction of Wood et al. (2014) that New World cutaneous leishmaniases should respond positively to biodiversity. In terms of mechanisms, more diverse communities of sylvatic wild mammal hosts may increase the pathogen pool to which humans are exposed (via infected sandflies) or may alter the cross-species transmission potential by increasing the abundance, susceptibility or contact rates of important hosts [7]. However, models including mammal biodiversity were similar in accuracy and predicted geographical disease extent to simpler models containing only abiotic climate and land use predictors. Although predictors were screened for collinearity by pair-wise correlation analysis between predictors, the mammal biodiversity predictors may be strongly related to particular combinations of climate or land-use predictors at a continental scale [62]. This collinearity between global climate and biodiversity layers is a key wider impediment to attempts to understand the relative effects of these factors on regional disease patterns.

Disease records are often biased towards more populous areas, regions with strong healthcare systems and funds for surveillance and control, and towards habitats or hosts in which primary disease impacts are felt (e.g. livestock, crops, people [63]). In contrast with the field of ecology [64], models of disease distributions rarely account for biases in disease recording despite their impacts on model accuracy and inference [53]. In their study of global leishmaniasis distribution [15], Piggott et al. built a spatial layer of leishmaniasis presence from expert opinion and used this to stratify pseudo-absence (and pseudo-presence) selection. We selected an approach which accounted for the process by which case data arise, namely the bias towards more populous areas with improved access to health care. Our results from preferentially selecting pseudo-absence data in more populous areas, highlight the importance of correcting for recording bias when modelling disease distributions [63]. In models that ignored these recording biases, the impact of urban land cover on the disease was over-estimated, and the disease distribution was vastly under-predicted, being extremely focal around urban areas (Figure A in S4 File, Table A in S4 File). Such focal urban distributions are implausible epidemiologically—initially, both diseases were linked to smaller, rural settlements near primary forest [28] and visceral leishmaniasis has only spread relatively recently into the margins of urban areas [25]. The predicted distributions from the bias-corrected models were more extensive and extended into rural areas and were therefore much more plausible.

Our study is the first to account for both climate change pathways and impacts of policy and socio-economic changes on land use in forecasts of future leishmaniasis distribution. Previous studies have projected recent climatic niches of leishmaniasis vectors and/or hosts onto future climates and inferred likely changes in human contact rates from the resulting patterns but have neglected future land use dynamics [38, 39, 65]. Our main prediction is that the continental-scale geographical extent of both forms of leishmaniasis are likely to contract under future climate change to 2050, but that key focal areas will be retained for both diseases. Interestingly we found that the differential thermal and moisture sensitivities of CL and VL, quantified by our recent past models, translated into divergent responses of these diseases to future climate pathways, with CL declining much more in distribution than VL, especially under strong climatic forcing. These results reinforce the importance of considering the sensitivity of vector-borne diseases to past and future environmental change for diseases and regions individually [4]. They highlight the importance of considering both temperature and moisture related aspects of future environmental change when dealing with diseases transmitted by arthropod vectors.

Projected disease impacts differed slightly between alternative socio-economic pathway and policy scenarios with VL in particular found to be less extensive under strong versus weak management for carbon, biodiversity and ecosystem services. This is expected given the lower explanatory power of land use versus climate predictors of past distributions found at this scale and does not indicate that future development and environmental strategies will have a low impact on leishmaniasis disease outcomes. Our framework could be fruitfully applied within eco-epidemiological regions or at narrower geographical scales (at which disease control decisions are often applied [4]) to link disease risk to land-use patterns and alternative development and environmental strategies.

Our correlative methods for predicting future disease patterns from recent past disease-environment relationships assume implicitly that these relationships will remain static into the future [66]. For an ecologically complex system like leishmaniasis, there are likely to be ecological and evolutionary means by which pathogens, vectors and reservoir hosts adapt to changing environments. This is illustrated by the recent adaptation of several sand fly vectors in different regions to peri-domestic habitats e.g. *Lutzomyia longipalis* and *L*. *cruzi* in Brazil [25] or new cropping systems [26] with knock-on impacts on human transmission. Mechanistic approaches such as explicit modelling of disease transmission dynamics to estimate R_0_ can accommodate non-linear and often opposing impacts of temperature on transmission cycle parameters. For zoonotic VL in the Mediterranean, R_0_, the risk of disease establishment once an infected host is introduced, has been mapped [67] by linking spatial climate and landscape driven models of sand fly vector distribution, to temperature impacts on sandfly mortality, biting and infection rates and dog-host parameters. These frameworks are very powerful but difficult to apply when vector and host roles and life cycles are poorly understood, as for the Leishmaniases in the Americas. They cannot easily accommodate wide-ranging environmental drivers unless responses to these drivers have been explicitly quantified. Recent novel correlative methods that infer vector-host-pathogen networks from species co-occurrence data [68] and link these to risk of human exposure particular land uses or zones [69] have a high potential for integration of climate and socio-economic scenarios. Such correlative methods should be underpinned by landscape-scale validation of vector-host-pathogen interactions and environmental responses.

Our study makes important advances towards frameworks for understanding disease disservices and services, their linkage to ecosystem characteristics, and for their prediction under alternative climate change pathways and socio-economic scenarios. A critical component was the consideration of spatially explicit land use and ecosystem patterns alongside climate at every step of the process and their linkages to alternative climate change mitigation options and policies. This is a pre-requisite of examining trade-offs between disease disservices/services and other ecosystem services, the need for which is increasingly being recognised in international policy [11].

## Supporting information

S1 File Environmental predictors of leishmaniasis distribution. (DOCX)

S2 File Trajectory of changes in climate and land use under alternative future socio-economic pathways, climate change pathways and policies. (DOCX)

S3 File Predicted distributions of leishmaniases when mammal richness is included or excluded from models. (DOCX)

S4 File Impact of correcting for recording bias on models of leishmaniasis distribution. (DOCX)

S5 File Predicted future extent of leishmaniasis under alternative climate pathways and socio-economic pathways. (DOCX)
