# Supplementary material for: How will climate change pathways and mitigation options alter incidence of vector-borne diseases? A framework for leishmaniasis in South and Meso-America
Source: PLoS One. 2017 Oct 11;12(10):e0183583. doi: 10.1371/journal.pone.0183583 (PMC5636069; doi:10.1371/journal.pone.0183583)
Supplement: S1 File — (DOCX) [file pone.0183583.s001.docx]

**S1. Environmental predictors of leishmaniasis distribution**

Landscape predictors: Table A indicates the land use classes available in the CLUE product. Several classes were dropped from the analysis because they were known apriori to be wholly unsuitable for sandflies (60 waterbodies, 53 bare desert, 70 ice & snow). An additional two classes were dropped since they were very infrequent across the ROBIN area, namely wetlands (61), and sparse grazed land (51). Availability and fragmentation of all other classes in the landscape was considered since sandlfy vectors involved in transmission have been linked to a wide range of habitat types including forest, cropland, shrubland and peri-urban areas [1, 2]. The CLUE (Conversion of Land Use and its Effects) model output layers were available at 1/10th of the study grid square resolution. Proportional cover as well as total edge and edge density of each class (measures of fragmentation) were calculated within the 5 arc minute cells (that each contained 100 CLUE pixels) for both the 2005 and 2050 time points. Considering collinearity within landscape metrics for the same class, proportional cover was highly correlated with both edge metrics except for the forest and crop classes. Thus, only for forest and crops were both edge metrics and proportional cover retained. Overall, 15 landscape metrics were considered (Table 2). The current percentage of irrigated land per cell was also obtained from Global irrigated area map data [3]. Elevation was extracted from Shuttle Radar Topography Mission data [4] and then summarised at the study grid square resolution. Elevation was only weakly correlated with climate and other landscape variables (r < 0.7).

Table A. Available land use classes in the CLUE (Conversion of Land Use and its Effects) landcover product [5]. The product is based on dynamic simulation of competition and interactions between land use types under different processes that may lead to land use change.

| Class  No. | Description | Starting age for simulation | Dynamic/Static |
| --- | --- | --- | --- |
| 10 | forest | 100 | Dynamic |
| 20 | shrubland | 50 | Dynamic |
| 21 | shrubland grazed | 5 | Dynamic |
| 30 | grassland | 50 | Dynamic |
| 31 | grassland grazed | 1 | Dynamic |
| 41 | cropland foodfeedfiber | 1 | Dynamic |
| 42 | cropland foodperennial | 5 | Dynamic |
| 43 | cropland energy | 5 | Dynamic |
| 50 | sparse | 10 | Static |
| 51 | sparse grazed | 5 | Static |
| 53 | bare or desert | 10 | Static |
| 60 | water | 10 | Static |
| 61 | wetland | 10 | Static |
| 62 | flooded/wetland forest | 10 | Static |
| 70 | ice & snow | 10 | Static |
| 80 | urban | 10 | Static |
| 90 | Abandoned (year >2005) | - | Dynamic |

References

1. Donalisio MR, Townsend Peterson A, Lemos Costa P, José da Silva F, França Valença H, Shaw JJ, et al. Microspatial Distributional Patterns of Vectors of Cutaneous Leishmaniasis in Pernambuco, Northeastern Brazil. Journal of Tropical Medicine. 2012;2012. doi: 10.1155/2012/642910.

2. Pinheiro MPG, Silva JHT, Cavalcanti KB, de Azevedo PRM, de Fátima Freire de Melo Ximenes M. Ecological interactions among phlebotomines (Diptera: Psychodidae) in an agroforestry environment of northeast Brazil. Journal of Vector Ecology. 2013;38(2):307-16. doi: 10.1111/j.1948-7134.2013.12045.x.

3. Siebert S, Henrich V, Frenken K, Burke J. Global Map of Irrigation Areas version 5. . In: Rheinische Friedrich-Wilhelms-University /Food and Agriculture Organization of the United Nations R, Italy, editor. Bonn, Germany2013.

4. Jarvis A, Reuter hI, Nelson A, Guevara E. Hole-filled SRTM for the globe Version 4. 2008.

5. van Eupen M, Cormont A, Kok K, Simoes M, Pereira S, Kolb M, et al. D2.2.1 Modelling land use change in Latin America. . 2014 Contract No.: Public report D2.2.1 from the EC ROBIN project.
