# Supplementary material for: How will climate change pathways and mitigation options alter incidence of vector-borne diseases? A framework for leishmaniasis in South and Meso-America
Source: PLoS One. 2017 Oct 11;12(10):e0183583. doi: 10.1371/journal.pone.0183583 (PMC5636069; doi:10.1371/journal.pone.0183583)
Supplement: S2 File — (DOCX) [file pone.0183583.s002.docx]

**S2. Trajectory of changes in climate and land use under alternative future socio-economic pathways, climate change pathways and policies**

Both representative concentration pathways (RCPs, RCP 2.6 and RCP 8.5), show increases in annual mean temperature (bio1) (obviously more pronounced in RCP 8.5), mean temperature seasonality (bio4) and maximum temperature of the warmest month (bio 5). Predicted increases in mean annual temperature are most pronounced in Brazil’s interior and along the north coast of south America (from east Venezuela to French Guiana) (Fig A). Mean temperature seasonality (bio4) and maximum temperature of the warmest month are also predicted to increase the most inland in Brazil. There is very little change in annual mean precipitation (bio12), slight increases in precipitation seasonality (bio 15) (particularly in RCP 8.5) and slight decreases in precipitation in the driest quarter (bio17) (Fig A).

The area of cropland increases in all scenarios but is most pronounced in the SSP5 scenarios, particularly SSP5s (Fig B,C, and D). The CroplandFood Perennial class occurs today largely along the east coast of Brazil and the west coast of Peru, Ecuador, Colombia and Venezuela and in central and southern Mexico (Fig B). The amount of edge of CroplandFood Perennial increases on average but also has a much wider spread of values across geographical area than in the current day suggesting fragmentation of natural habitat habitats by crops (Fig C). Increases in cover and total area are most pronounced in Mexico, Peru, Ecuador and Colombia. The CroplandFoodFiber class is currently found mostly in eastern Brazil and throughout Mexico (Fig. D). Predicted increases in this cover type are most pronounced in central and southern Mexico and in central Brazil and along the edges of the amazon forest. The amount of forest cover is reduced on average in the SSP 5 land use scenarios but is similar to current day values in SSP 1. Predicted declines in forest cover are most pronounced (Fig E) in central Brazil, and northern Brazil on the fringes of the amazon forest and in Peru, Ecuador and Colombia, these areas mirroring those where cropland increases in cover. The amount of urban cover increases slightly in SSP 5 compared to the current day but is largely static into the future. Changes in the amount of irrigated land were not encompassed in these land cover scenarios.


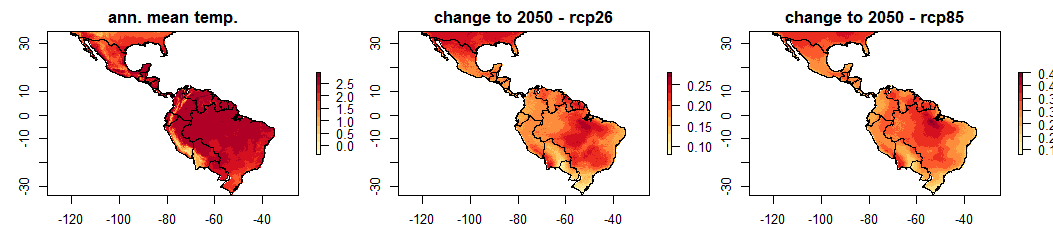


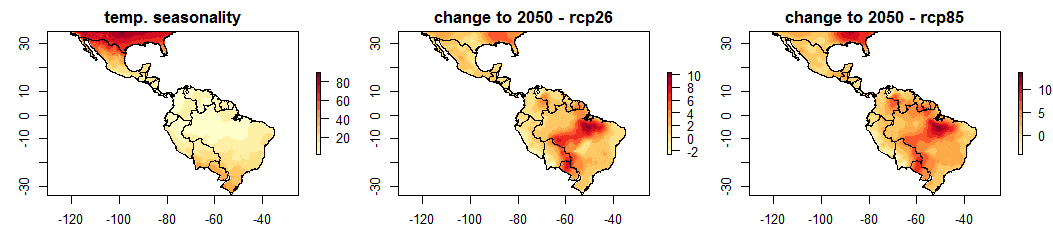


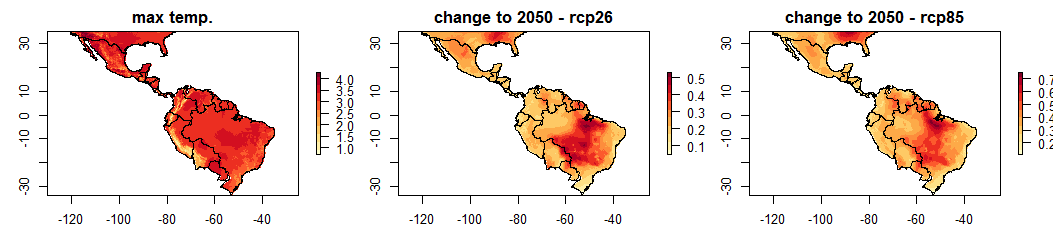


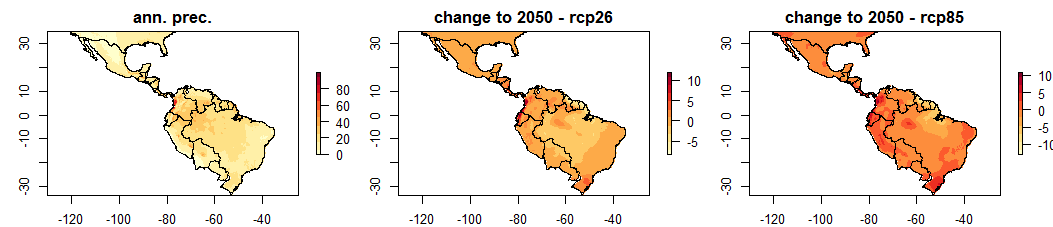

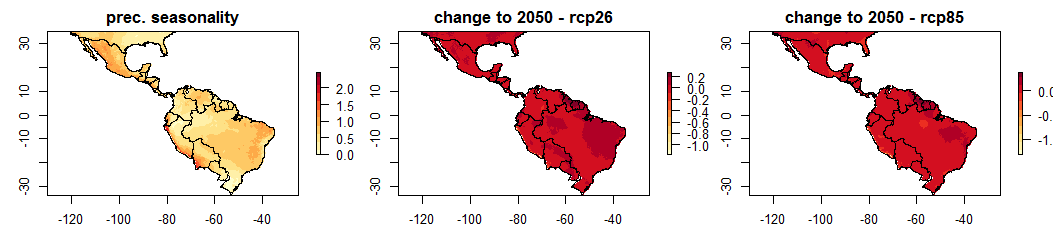

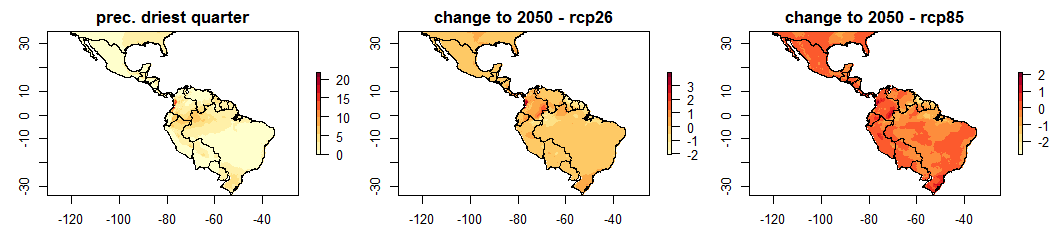


Fig A. Maps of key climate predictors - recent past (2005) values and absolute change in these predictors to 2050 under different RCPs.


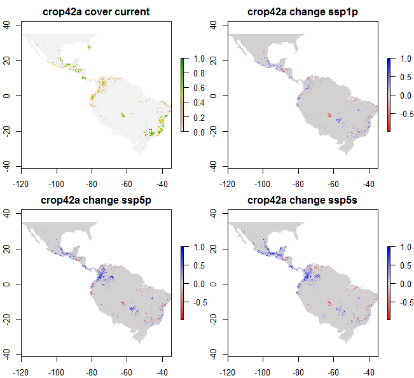


Fig. B. Maps of key landcover predictors: Crop – food perennial type cover in the recent past (2005) values and absolute change in these predictors to 2050 under different land use change scenarios.


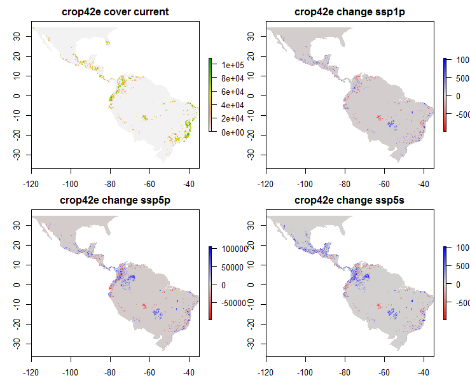


Fig. C. Maps of key landcover predictors: Crop – food perennial type total edge in the recent past (2005) values and absolute change in these predictors to 2050 under different land use change scenarios.


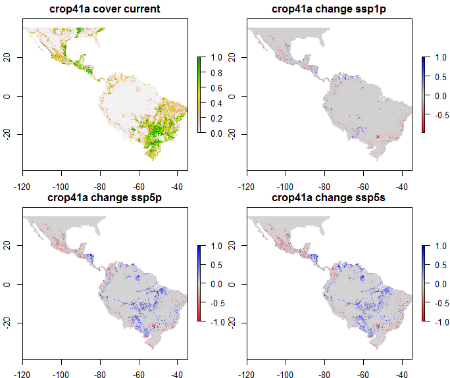


Fig. D. Maps of key landcover predictors: Crop – food fibre type cover in the recent past (2005) values and absolute change in these predictors to 2050 under different land use change scenarios.


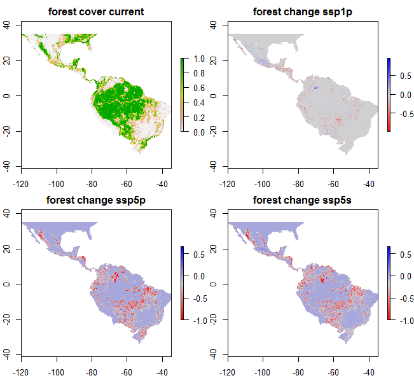


Fig. E. Maps of key landcover predictors: forest cover in the recent past (2005) values and absolute change in these predictors to 2050 under different land use change scenarios.
