# Supplementary material for: How will climate change pathways and mitigation options alter incidence of vector-borne diseases? A framework for leishmaniasis in South and Meso-America
Source: PLoS One. 2017 Oct 11;12(10):e0183583. doi: 10.1371/journal.pone.0183583 (PMC5636069; doi:10.1371/journal.pone.0183583)
Supplement: S3 File — (DOCX) [file pone.0183583.s003.docx]

**S3. Predicted distributions of leishmaniases when mammal richness is included or excluded from models.**


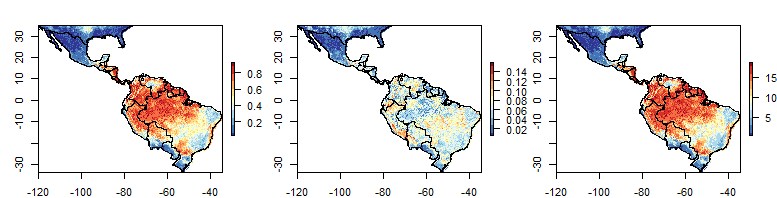


ntimes

abiotic

sd abiotic

mean abiotic


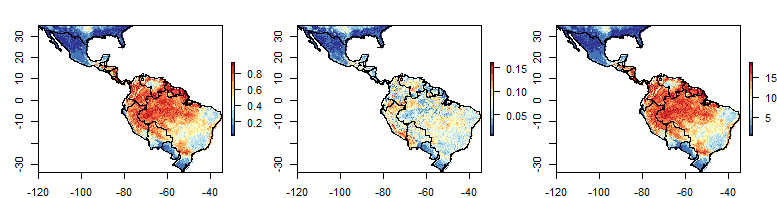


ntimes

all mamm

sd

all mamm

mean

all mamm


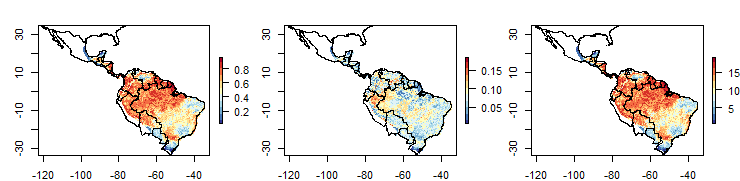


ntimes

ord mamm

mean

ord mamm

sd

ord mamm

Fig A. Predicted distributions of cutaneous leishmaniasis when mammal richness is included or excluded from models. Columns show mean (mean), standard deviation (sd) of relative probability of presence and sum of times (ntimes) predicted to be present across 20 runs. Row 1 contains results from abiotic only models (abiotic), row 2 from abiotic + all mammal richness models (all mamm), row 3 from abiotic + richness of mammal order models (ord mamm).


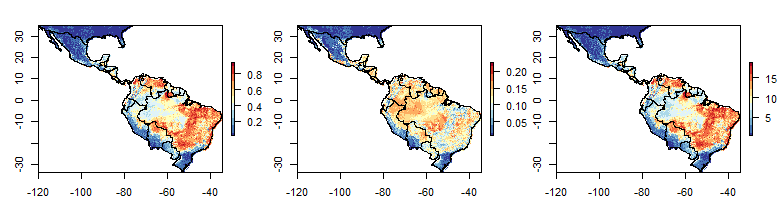


ntimes

Abiotic

sd Abiotic

mean Abiotic


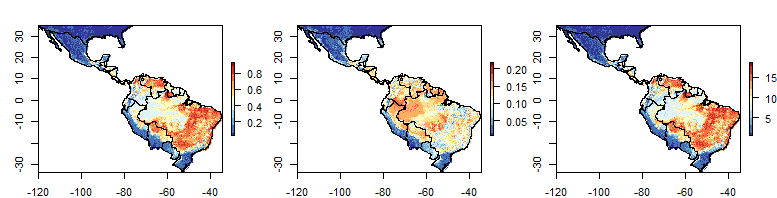


ntimes

all mamm

sd

all mamm

mean

all mamm


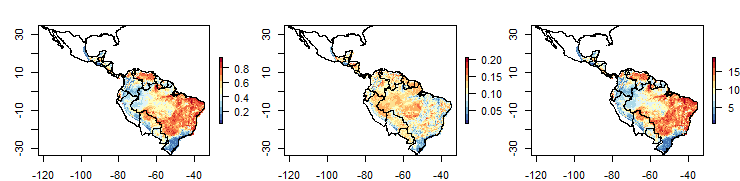


ntimes

ord mamm

sd

ord mamm

mean

ord mamm

Fig B. Predicted distributions of visceral leishmaniasis when mammal richness is included or excluded from models. Columns show mean (mean), standard deviation (sd) of relative probability of presence and sum of times (ntimes) predicted to be present across 20 runs. Row 1 contains results from abiotic only models (abiotic), row 2 from abiotic + all mammal richness models (all mamm), row 3 from abiotic + richness of mammal order models (ord mamm).
