# Supplementary material for: How will climate change pathways and mitigation options alter incidence of vector-borne diseases? A framework for leishmaniasis in South and Meso-America
Source: PLoS One. 2017 Oct 11;12(10):e0183583. doi: 10.1371/journal.pone.0183583 (PMC5636069; doi:10.1371/journal.pone.0183583)
Supplement: S4 File — (DOCX) [file pone.0183583.s004.docx]

**S4. Impact of correcting for recording bias on models of leishmaniasis distribution**

Climate effects dominate the corrected models (see main text) with land use factors having secondary importance. In the uncorrected models, for both disease forms urban land cover is the most important predictor rather than temperature/precipitation effects. The uncorrected models vastly under-predict the distributions of the disease forms compared to the corrected models (Fig. S5). The correct models are consistent with the predicted distribution of cases and expert opinion from other studies (see discussion).

Table A. Percentage contribution of top ten ranked predictors to models of visceral and cutaneous leishmaniasis that were not corrected for the biased recording of disease case data in more populous areas (averaged across 20 sub-models).

| **visceral leishmaniasis** | % contribution | | **cutaneous leishmaniasis** | % contribution | |
| --- | --- | --- | --- | --- | --- |
| Predictor | mean | sd | Predictor | mean | sd |
| Urban land class cover | 36.9 | 4.4 | Urban cover | 12.2 | 2.1 |
| Temperature seasonality | 7.1 | 1.7 | Temperature seasonality | 10.0 | 1.1 |
| precipitation annual mean | 6.4 | 1.4 | precipitation seasonality | 8.2 | 1.2 |
| temperature annual mean | 4.6 | 1.4 | Max temp. warmest month | 8.0 | 1.7 |
| Irrigated land area | 4.4 | 1.5 | precipitation annual mean | 7.0 | 0.4 |
| Max temp. warmest month | 4.3 | 0.8 | Cropland foodPerennial edge | 6.2 | 1.6 |
| precipitation seasonality | 4.1 | 0.7 | elevation | 5.9 | 0.8 |
| elevation | 4.0 | 0.8 | Forest cover | 5.8 | 1.0 |
| Cropland foodPerennial edge | 4.0 | 1.0 | precipitation driest quarter | 5.1 | 0.6 |
| Cropland FoodFeedFiber cover | 3.4 | 0.4 | Cropland FoodFeedFiber cover | 4.9 | 0.3 |


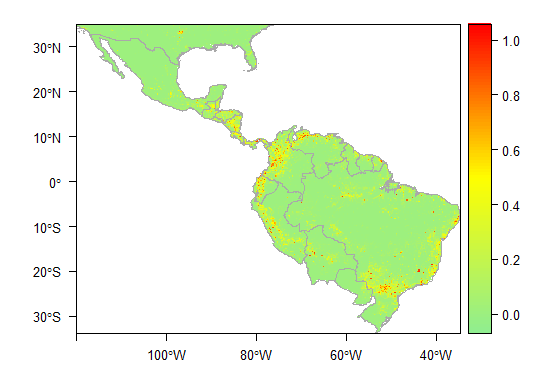

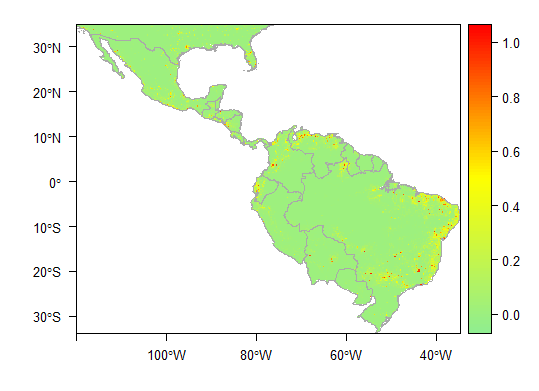


Fig A. Predicted relative probability of presence of (a) CL and (b) VL from uncorrected BRT models.

**S5. Predicted future extent of leishmaniasis under alternative climate pathways and socio-economic pathways.**
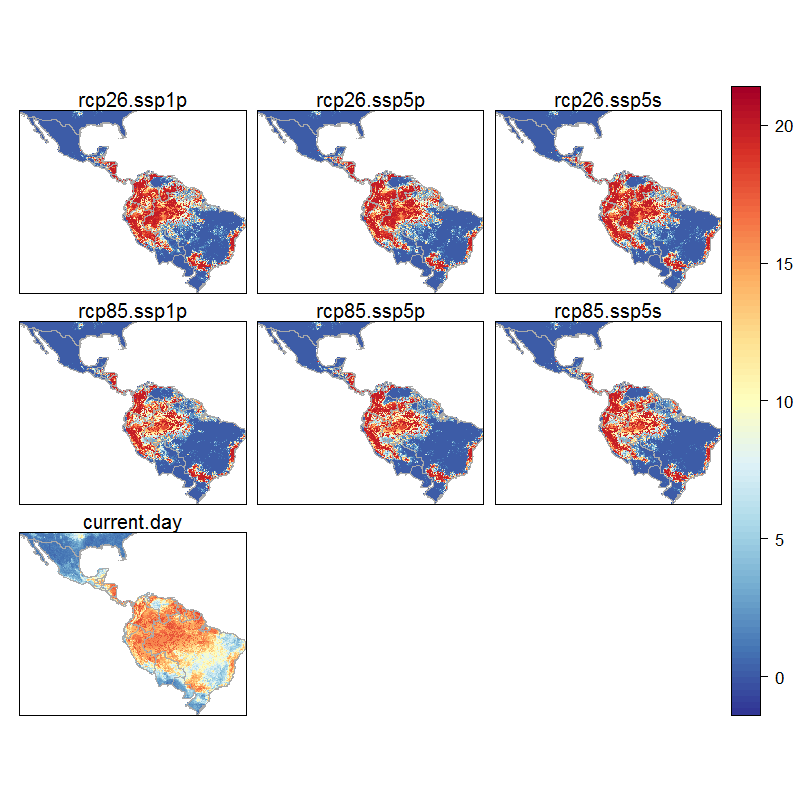


Fig A. Predicted future extent of cutaneous leishmaniasis under alternative climate pathways and socio-economic pathways. Colour scale indicates the number of times a pixel is predicted as present across 20 model runs, from 0 in green up to 20 in red.


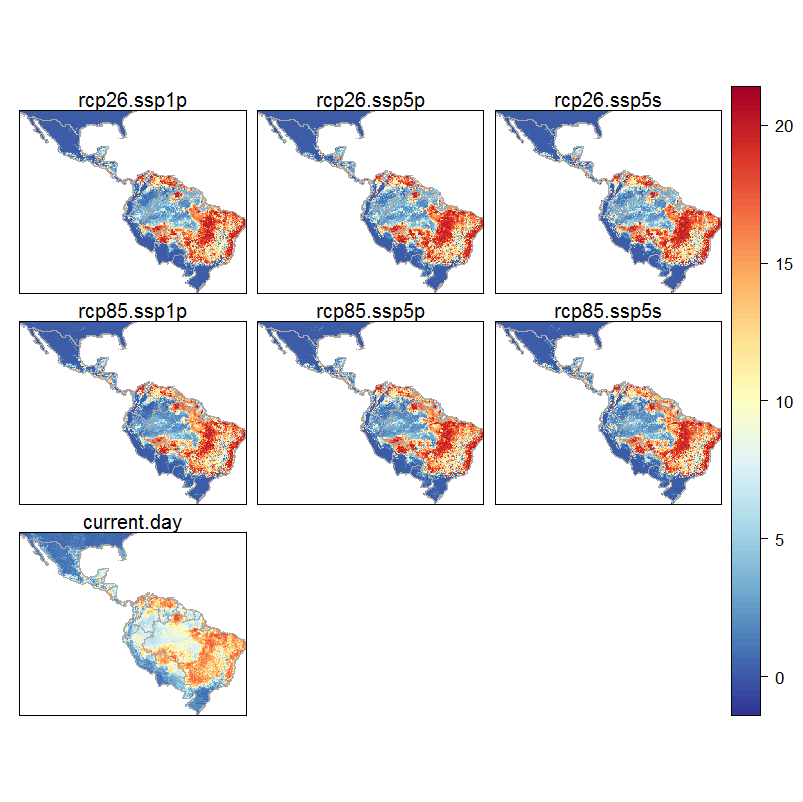


Fig. B Predicted future extent of visceral leishmaniasis under alternative climate pathways and socio-economic pathways. Colour scale indicates the number of times a pixel is predicted as present across 20 model runs, from 0 in green up to 20 in red.
