# Supplementary material for: How will climate change pathways and mitigation options alter incidence of vector-borne diseases? A framework for leishmaniasis in South and Meso-America
Source: PLoS One. 2017 Oct 11;12(10):e0183583. doi: 10.1371/journal.pone.0183583 (PMC5636069; doi:10.1371/journal.pone.0183583)
Supplement: S5 File — (DOCX) [file pone.0183583.s005.docx]

**Supplementary information**

**S1. Environmental predictors of leishmaniasis distribution**

Landscape predictors: Table S1 indicates the land use classes available in the CLUE product. Several classes were dropped from the analysis because they were known apriori to be wholly unsuitable for sandflies (60 waterbodies, 53 bare desert, 70 ice & snow). An additional two classes were dropped since they were very infrequent across the ROBIN area, namely wetlands (61), and sparse grazed land (51). Availability and fragmentation of all other classes in the landscape was considered since sandlfy vectors involved in transmission have been linked to a wide range of habitat types including forest, cropland, shrubland and peri-urban areas [1, 2]. The CLUE (Conversion of Land Use and its Effects) model output layers were available at 1/10th of the study grid square resolution. Proportional cover as well as total edge and edge density of each class (measures of fragmentation) were calculated within the 5 arc minute cells (that each contained 100 CLUE pixels) for both the 2005 and 2050 time points. Considering collinearity within landscape metrics for the same class, proportional cover was highly correlated with both edge metrics except for the forest and crop classes. Thus, only for forest and crops were both edge metrics and proportional cover retained. Overall, 15 landscape metrics were considered (Table 2). The current percentage of irrigated land per cell was also obtained from Global irrigated area map data [3]. Elevation was extracted from Shuttle Radar Topography Mission data [4] and then summarised at the study grid square resolution. Elevation was only weakly correlated with climate and other landscape variables (r < 0.7).

Table S1. Available land use classes in the CLUE (Conversion of Land Use and its Effects) landcover product [5]. The product is based on dynamic simulation of competition and interactions between land use types under different processes that may lead to land use change.

| Class  No. | Description | Starting age for simulation | Dynamic/Static |
| --- | --- | --- | --- |
| 10 | forest | 100 | Dynamic |
| 20 | shrubland | 50 | Dynamic |
| 21 | shrubland grazed | 5 | Dynamic |
| 30 | grassland | 50 | Dynamic |
| 31 | grassland grazed | 1 | Dynamic |
| 41 | cropland foodfeedfiber | 1 | Dynamic |
| 42 | cropland foodperennial | 5 | Dynamic |
| 43 | cropland energy | 5 | Dynamic |
| 50 | sparse | 10 | Static |
| 51 | sparse grazed | 5 | Static |
| 53 | bare or desert | 10 | Static |
| 60 | water | 10 | Static |
| 61 | wetland | 10 | Static |
| 62 | flooded/wetland forest | 10 | Static |
| 70 | ice & snow | 10 | Static |
| 80 | urban | 10 | Static |
| 90 | Abandoned (year >2005) | - | Dynamic |

References

1. Donalisio MR, Townsend Peterson A, Lemos Costa P, José da Silva F, França Valença H, Shaw JJ, et al. Microspatial Distributional Patterns of Vectors of Cutaneous Leishmaniasis in Pernambuco, Northeastern Brazil. Journal of Tropical Medicine. 2012;2012. doi: 10.1155/2012/642910.

2. Pinheiro MPG, Silva JHT, Cavalcanti KB, de Azevedo PRM, de Fátima Freire de Melo Ximenes M. Ecological interactions among phlebotomines (Diptera: Psychodidae) in an agroforestry environment of northeast Brazil. Journal of Vector Ecology. 2013;38(2):307-16. doi: 10.1111/j.1948-7134.2013.12045.x.

3. Siebert S, Henrich V, Frenken K, Burke J. Global Map of Irrigation Areas version 5. . In: Rheinische Friedrich-Wilhelms-University /Food and Agriculture Organization of the United Nations R, Italy, editor. Bonn, Germany2013.

4. Jarvis A, Reuter hI, Nelson A, Guevara E. Hole-filled SRTM for the globe Version 4. 2008.

5. van Eupen M, Cormont A, Kok K, Simoes M, Pereira S, Kolb M, et al. D2.2.1 Modelling land use change in Latin America. . 2014 Contract No.: Public report D2.2.1 from the EC ROBIN project.

**S2. Trajectory of changes in climate and land use under alternative future socio-economic pathways, climate change pathways and policies**

Both representative concentration pathways (RCPs, RCP 2.6 and RCP 8.5), show increases in annual mean temperature (bio1) (obviously more pronounced in RCP 8.5), mean temperature seasonality (bio4) and maximum temperature of the warmest month (bio 5). Predicted increases in mean annual temperature are most pronounced in Brazil’s interior and along the north coast of south America (from east Venezuela to French Guiana) (Fig. S2.1). Mean temperature seasonality (bio4) and maximum temperature of the warmest month are also predicted to increase the most inland in Brazil. There is very little change in annual mean precipitation (bio12), slight increases in precipitation seasonality (bio 15) (particularly in RCP 8.5) and slight decreases in precipitation in the driest quarter (bio17) (Fig. S2.1).

The area of cropland increases in all scenarios but is most pronounced in the SSP5 scenarios, particularly SSP5s (Fig.S2.2b, c, d). The CroplandFood Perennial class occurs today largely along the east coast of Brazil and the west coast of Peru, Ecuador, Colombia and Venezuela and in central and southern Mexico. The amount of edge of CroplandFood Perennial increases on average but also has a much wider spread of values across geographical area than in the current day suggesting fragmentation of natural habitat habitats by crops. Increases in cover and total area are most pronounced in Mexico, Peru, Ecuador and Colombia. The CroplandFoodFiber class is currently found mostly in eastern Brazil and throughout Mexico. Predicted increases in this cover type are most pronounced in central and southern Mexico and in central Brazil and along the edges of the amazon forest. The amount of forest cover is reduced on average in the SSP 5 land use scenarios but is similar to current day values in SSP 1. Predicted declines in forest cover are most pronounced (Fig. s2.2a) in central Brazil, and northern Brazil on the fringes of the amazon forest and in Peru, Ecuador and Colombia, these areas mirroring those where cropland increases in cover. The amount of urban cover increases slightly in SSP 5 compared to the current day but is largely static into the future. Changes in the amount of irrigated land were not encompassed in these land cover scenarios.


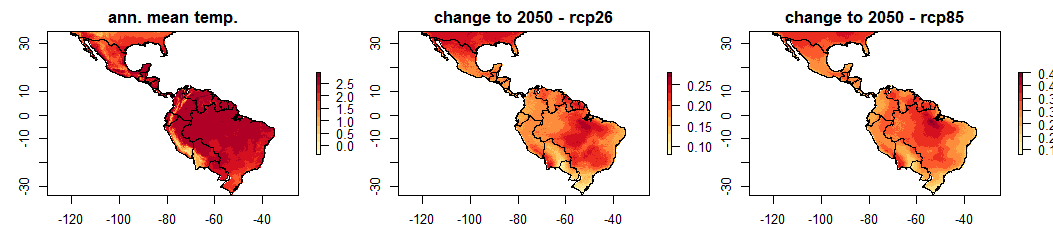


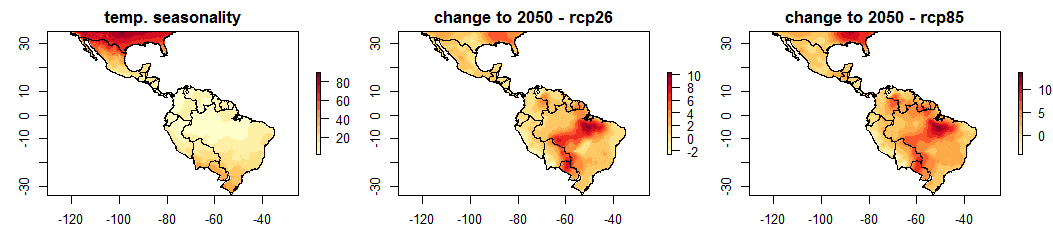


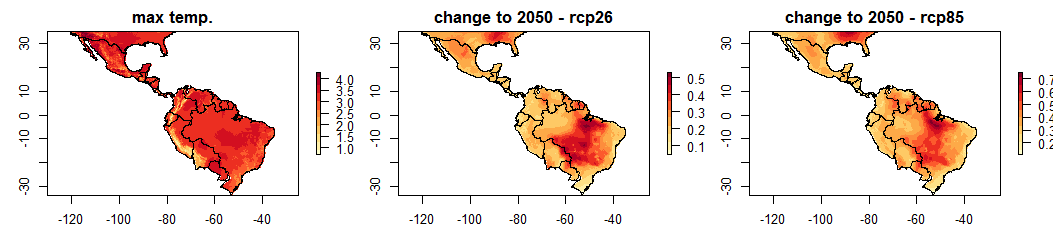


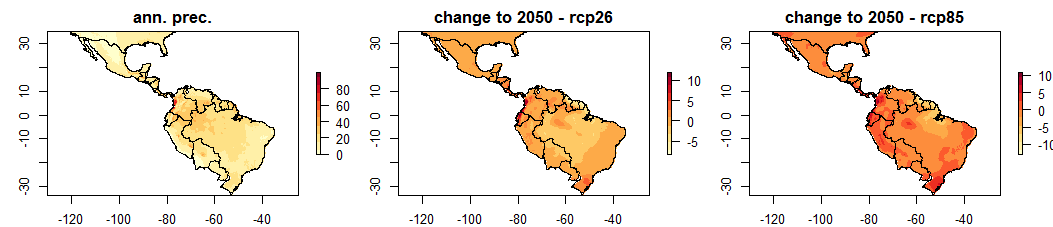

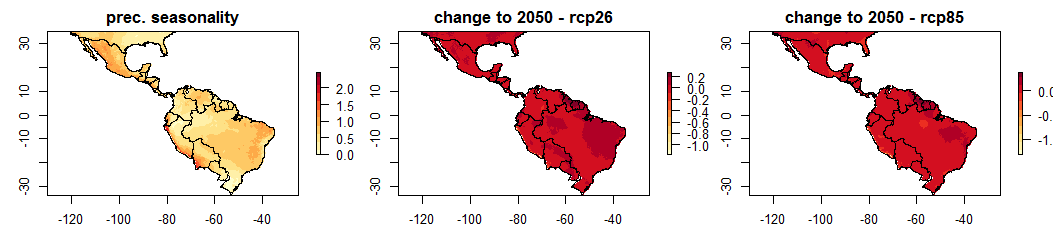

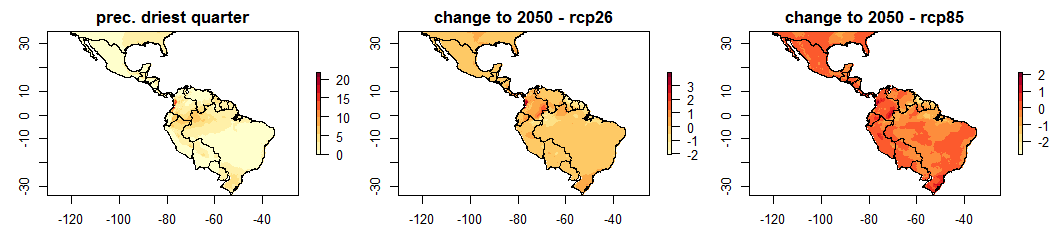


Fig. S2.1. Maps of key climate predictors - recent past (2005) values and absolute change in these predictors to 2050 under different RCPs.

1. Crop – food perennial type cover


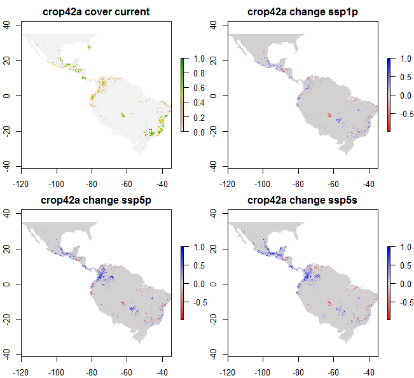


1. Crop – food perennial type total edge


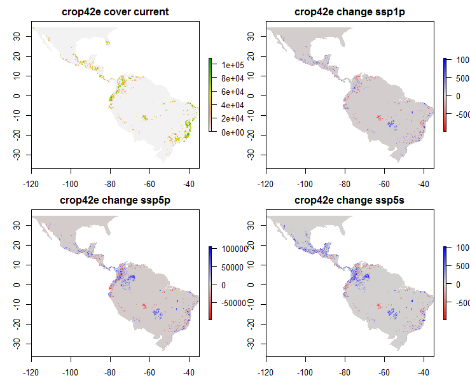


1. Crop – food fibre type cover


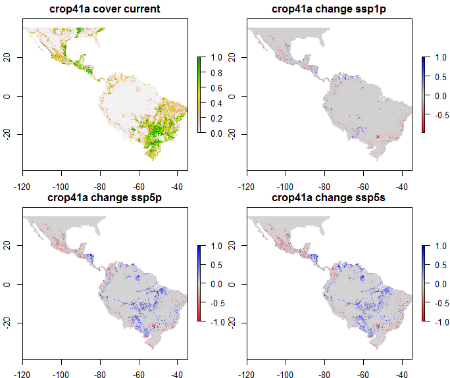


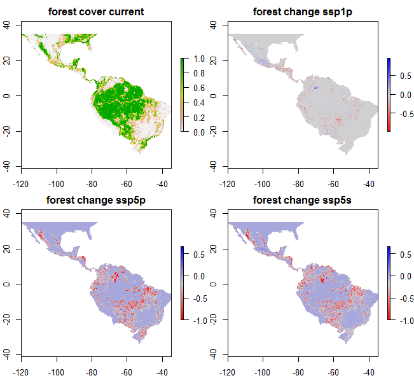


Fig. S2.2. Maps of key landcover predictors - recent past (2005) values and absolute change in these predictors to 2050 under different land use change scenarios

**S3. Predicted distributions of leishmaniases when mammal richness is included or excluded from models.**


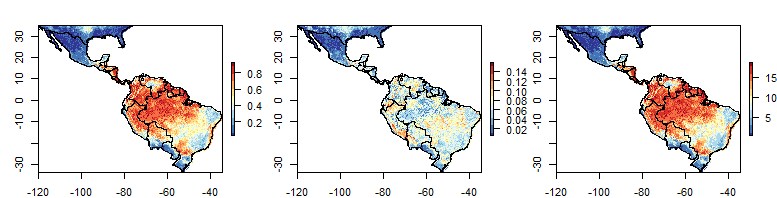


ntimes

abiotic

sd abiotic

mean abiotic


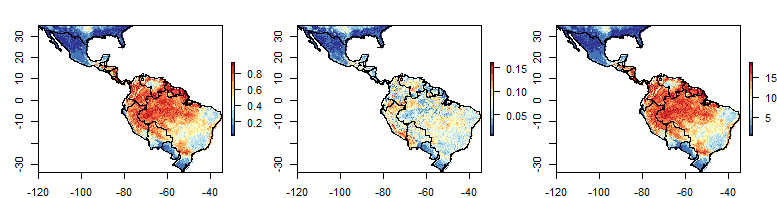


ntimes

all mamm

sd

all mamm

mean

all mamm


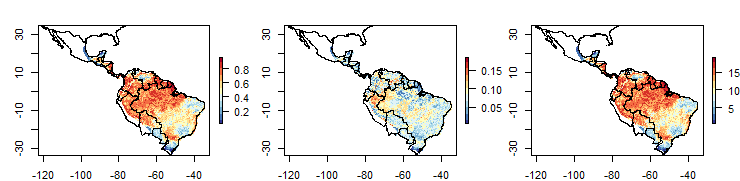


ntimes

ord mamm

mean

ord mamm

sd

ord mamm

Fig. S3.1. Predicted distributions of cutaneous leishmaniasis when mammal richness is included or excluded from models. Columns show mean (mean), standard deviation (sd) of relative probability of presence and sum of times (ntimes) predicted to be present across 20 runs. Row 1 contains results from abiotic only models (abiotic), row 2 from abiotic + all mammal richness models (all mamm), row 3 from abiotic + richness of mammal order models (ord mamm).


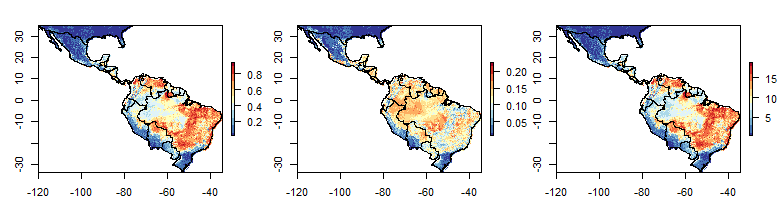


ntimes

Abiotic

sd Abiotic

mean Abiotic


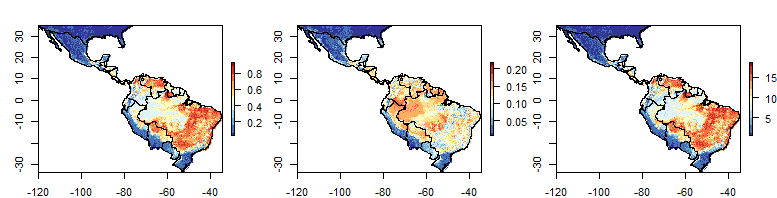


ntimes

all mamm

sd

all mamm

mean

all mamm


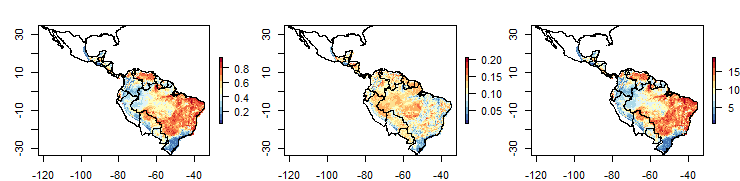


ntimes

ord mamm

sd

ord mamm

mean

ord mamm

Fig. S3.2. Predicted distributions of visceral leishmaniasis when mammal richness is included or excluded from models. Columns show mean (mean), standard deviation (sd) of relative probability of presence and sum of times (ntimes) predicted to be present across 20 runs. Row 1 contains results from abiotic only models (abiotic), row 2 from abiotic + all mammal richness models (all mamm), row 3 from abiotic + richness of mammal order models (ord mamm).

**S4. Impact of correcting for recording bias on models of leishmaniasis distribution**

Climate effects dominate the corrected models (see main text) with land use factors having secondary importance. In the uncorrected models, for both disease forms urban land cover is the most important predictor rather than temperature/precipitation effects. The uncorrected models vastly under-predict the distributions of the disease forms compared to the corrected models (Fig. S5). The correct models are consistent with the predicted distribution of cases and expert opinion from other studies (see discussion).

Table S4. Percentage contribution of top ten ranked predictors to models of visceral and cutaneous leishmaniasis that were not corrected for the biased recording of disease case data in more populous areas (averaged across 20 sub-models).

| **visceral leishmaniasis** | % contribution | | **cutaneous leishmaniasis** | % contribution | |
| --- | --- | --- | --- | --- | --- |
| Predictor | mean | sd | Predictor | mean | sd |
| Urban land class cover | 36.9 | 4.4 | Urban cover | 12.2 | 2.1 |
| Temperature seasonality | 7.1 | 1.7 | Temperature seasonality | 10.0 | 1.1 |
| precipitation annual mean | 6.4 | 1.4 | precipitation seasonality | 8.2 | 1.2 |
| temperature annual mean | 4.6 | 1.4 | Max temp. warmest month | 8.0 | 1.7 |
| Irrigated land area | 4.4 | 1.5 | precipitation annual mean | 7.0 | 0.4 |
| Max temp. warmest month | 4.3 | 0.8 | Cropland foodPerennial edge | 6.2 | 1.6 |
| precipitation seasonality | 4.1 | 0.7 | elevation | 5.9 | 0.8 |
| elevation | 4.0 | 0.8 | Forest cover | 5.8 | 1.0 |
| Cropland foodPerennial edge | 4.0 | 1.0 | precipitation driest quarter | 5.1 | 0.6 |
| Cropland FoodFeedFiber cover | 3.4 | 0.4 | Cropland FoodFeedFiber cover | 4.9 | 0.3 |


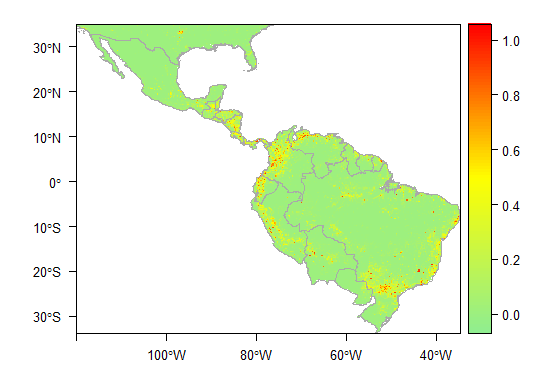

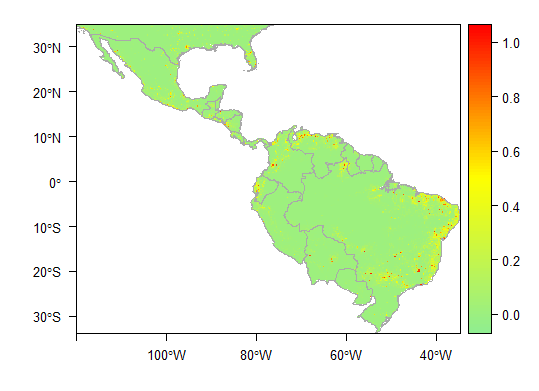


Fig. S4. Predicted relative probability of presence of (a) CL and (b) VL from uncorrected BRT models.

**S5. Predicted future extent of leishmaniasis under alternative climate pathways and socio-economic pathways.**
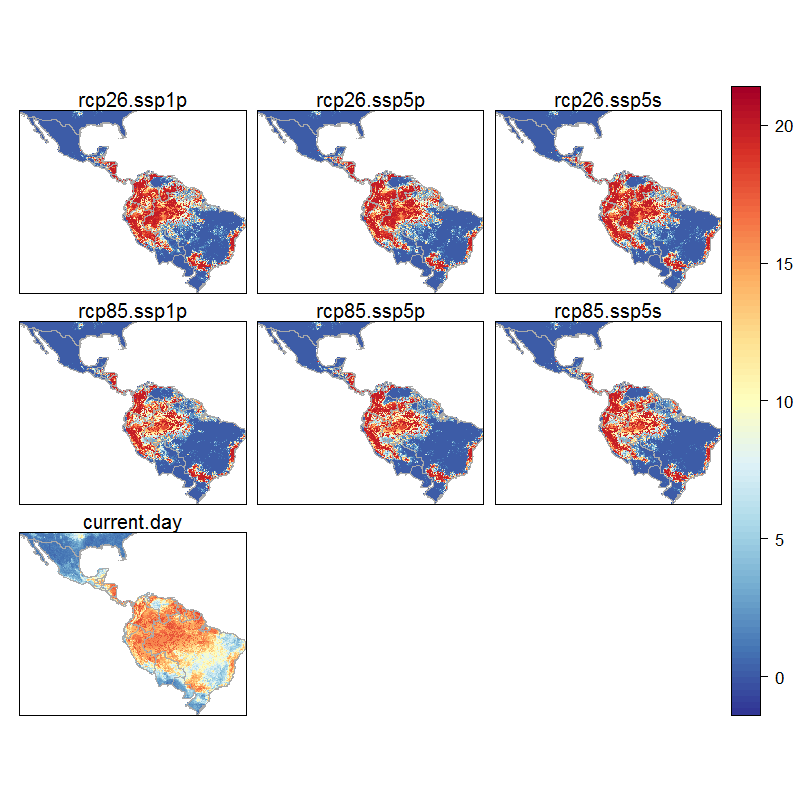


Fig. S5.1. Predicted future extent of cutaneous leishmaniasis under alternative climate pathways and socio-economic pathways. Colour scale indicates the number of times a pixel is predicted as present across 20 model runs, from 0 in green up to 20 in red.


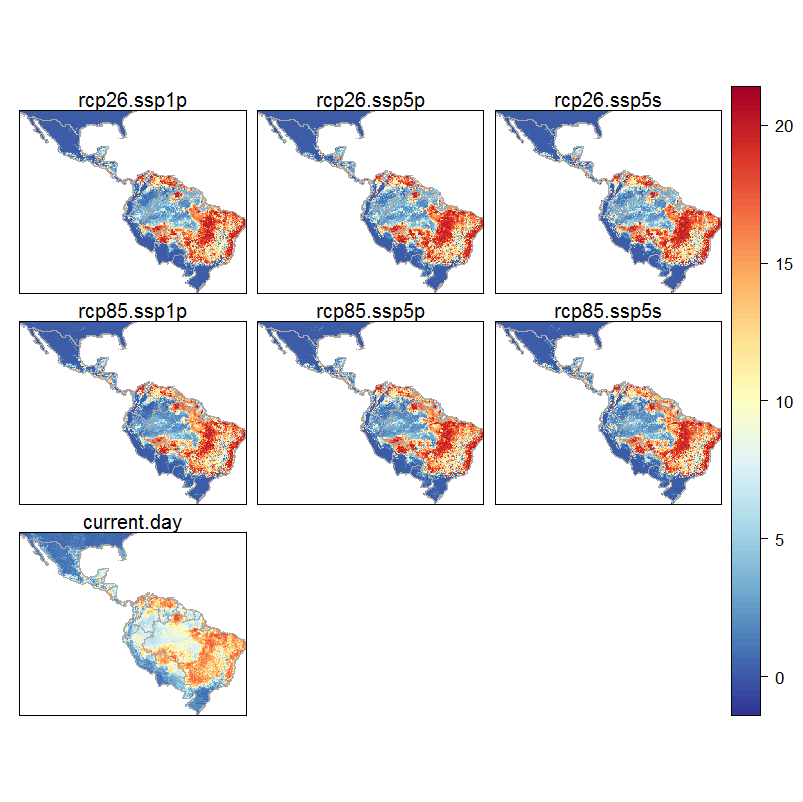


Fig. S5.2. Predicted future extent of visceral leishmaniasis under alternative climate pathways and socio-economic pathways. Colour scale indicates the number of times a pixel is predicted as present across 20 model runs, from 0 in green up to 20 in red.
